# Microbial consortium assembly and functional analysis via isotope labelling and single-cell manipulation of polycyclic aromatic hydrocarbon degraders

**DOI:** 10.1093/ismejo/wrae115

**Published:** 2024-06-24

**Authors:** Jibing Li, Chunling Luo, Xixi Cai, Dayi Zhang, Guoqing Guan, Bei Li, Gan Zhang

**Affiliations:** State Key Laboratory of Organic Geochemistry and Guangdong-Hong Kong-Macao Joint Laboratory for Environmental Pollution and Control, Guangzhou Institute of Geochemistry, Chinese Academy of Sciences, Guangzhou 510640, China; College of Resources and Environment, University of Chinese Academy of Sciences, Beijing 100039, China; State Key Laboratory of Organic Geochemistry and Guangdong-Hong Kong-Macao Joint Laboratory for Environmental Pollution and Control, Guangzhou Institute of Geochemistry, Chinese Academy of Sciences, Guangzhou 510640, China; College of Resources and Environment, University of Chinese Academy of Sciences, Beijing 100039, China; Guangdong Key Laboratory of Ornamental Plant Germplasm Innovation and Utilization, Environmental Horticulture Research Institute, Guangdong Academy of Agricultural Sciences, Guangzhou 510640, China; Key Laboratory of Groundwater Resources and Environment, Ministry of Education, Jilin University, Changchun 130012, China; College of New Energy and Environment, Jilin University, Changchun 130021, China; State Key Laboratory of Organic Geochemistry and Guangdong-Hong Kong-Macao Joint Laboratory for Environmental Pollution and Control, Guangzhou Institute of Geochemistry, Chinese Academy of Sciences, Guangzhou 510640, China; College of Resources and Environment, University of Chinese Academy of Sciences, Beijing 100039, China; State Key Lab of Applied Optics, Changchun Institute of Optics, Fine Mechanics and Physics, Chinese Academy of Sciences, Changchun 130033, China; Jilin Province Raman Technology Engineering Research Center, HOOKE Instruments Ltd., Changchun 130033, China; State Key Laboratory of Organic Geochemistry and Guangdong-Hong Kong-Macao Joint Laboratory for Environmental Pollution and Control, Guangzhou Institute of Geochemistry, Chinese Academy of Sciences, Guangzhou 510640, China; College of Resources and Environment, University of Chinese Academy of Sciences, Beijing 100039, China

**Keywords:** *in situ* functional microbial consortia, Raman-activated cell sorting, stable-isotope probing, genome-directed cultivation, soil phenanthrene-degrading bacteria, single-cell genomic sequencing

## Abstract

Soil microbial flora constitutes a highly diverse and complex microbiome on Earth, often challenging to cultivation, with unclear metabolic mechanisms *in situ*. Here, we present a pioneering concept for the *in situ* construction of functional microbial consortia (FMCs) and introduce an innovative method for creating FMCs by utilizing phenanthrene as a model compound to elucidate their *in situ* biodegradation mechanisms. Our methodology involves single-cell identification, sorting, and culture of functional microorganisms, resulting in the formation of a precise *in situ* FMC. Through Raman-activated cell sorting–stable-isotope probing, we identified and isolated phenanthrene-degrading bacterial cells from *Achromobacter* sp. and *Pseudomonas* sp., achieving precise and controllable *in situ* consortia based on genome-guided cultivation. Our *in situ* FMC outperformed conventionally designed functional flora when tested in real soil, indicating its superior phenanthrene degradation capacity. We revealed that microorganisms with high degradation efficiency isolated through conventional methods may exhibit pollutant tolerance but lack actual degradation ability in natural environments. This finding highlights the potential to construct FMCs based on thorough elucidation of *in situ* functional degraders, thereby achieving sustained and efficient pollutant degradation. Single-cell sequencing linked degraders with their genes and metabolic pathways, providing insights regarding the construction of *in situ* FMCs. The consortium *in situ* comprising microorganisms with diverse phenanthrene metabolic pathways might offer distinct advantages for enhancing phenanthrene degradation efficiency, such as the division of labour and cooperation or communication among microbial species. Our approach underscores the importance of *in situ*, single-cell precision identification, isolation, and cultivation for comprehensive bacterial functional analysis and resource exploration, which can extend to investigate MFCs in archaea and fungi, clarifying FMC construction methods for element recycling and pollutant transformation in complex real-world ecosystems.

## Introduction

Microorganisms play pivotal roles in Earth’s biogeochemical cycles and are essential to the degradation and transformation of pollutants [1, 2]. They act as biological mediators within ecosystems, significantly contributing to vital ecological processes such as nutrient cycling, organic matter decomposition, and soil remediation [3]. Notably, during environmental pollutant degradation, functional microorganisms often operate in the form of communities, rather than individual microorganisms [4]. However, in real-world environments, it is exceedingly difficult to characterize microbial communities that are truly capable of pollutant degradation. Historically, researchers have utilized cultivation-dependent methods to isolate and cultivate functional bacteria, obtaining insights into their biodegradation mechanisms through analyses of genetic and metabolic pathways [5, 6]. Although this approach is advantageous, it has limitations, particularly with regard to uncultured microorganisms [7]. Moreover, bacteria that can be efficiently cultivated in the laboratory may not necessarily represent the microorganisms responsible for the *in situ* degradation in natural ecosystems or reflect the interactions among microbes [8, 9]. This is where cultivation-independent techniques prove invaluable. For instance, stable-isotope probing (SIP) utilizes labelled substrates to trace functional microbial groups involved in the *in situ* biodegradation of organic pollutants in complex microbial communities, linking their identities to functions [10, 11]. Although the SIP method can elucidate the ecological roles and degradation mechanisms of the functional community, it is limited to the identification *in situ* at the community level; it cannot achieve the isolation and cultivation of specific functional bacteria, establish genotype–function correlations, or reconstruct the functional microbial consortia (FMCs) in real environments (i.e. *in situ* functional microorganisms) [12]. This shortcoming represents a key research challenge and technical bottleneck in the field of environmental microbiology.

The advent of Raman-activated cell sorting (RACS)-SIP offers the potential to resolve these research challenges related to functional bacteria *in situ* and their degradation mechanisms in contaminated soils [13, 14]. This cutting-edge method utilizes the shift in Raman bands of isotopically labelled biomolecules within microbial cells to identify and sort specific functional microbial cells. Thus far, this method has primarily been employed to characterize antibiotic-resistant and carbon-fixing bacteria [14–16]. Advanced methods of this nature are rarely utilized to investigate *in situ* functional bacteria degrading common soil organic pollutants such as polycyclic aromatic hydrocarbons (PAHs). Additionally, substantial challenges persist in the cultivation of single microbial cells. Considering that analyses of the genetic and metabolic traits of microorganisms can yield essential insights into their growth requirements, single-cell genomic analysis can be used to acquire the genomes of target functional microbial cells, infer their genetic traits, and predict their culture medium requirements, thus facilitating their cultivation [17, 18]. To date, only one study has implemented RACS-SIP and genome-directed cultivation to identify, sort, and cultivate active BTEX degrading microorganisms at the single-cell level [3]. This method has not yet been applied to studies regarding the degradation of other organic pollutants.

Thus, using RACS-SIP coupled with the genome sequencing technology described above, various types of functional microbial cells can be identified, acquired, and cultured [3, 18]. This process assists with the reconstruction of functional microbial communities that genuinely contribute to organic pollutant degradation in the *in situ* environment. Currently, the construction of functional degrading bacterial communities primarily relies on selective pressure screening of bacterial communities from natural environments or the use of efficient organic pollutant-degrading bacteria to generate synthetic microbial communities [19, 20]. However, no research has established an *in situ* FMC on the basis of functional microorganisms collected in real environments. The emergence of such a technique could expedite the acquisition of active microorganisms participating in the degradation of actual environmental pollutants and the construction of *in situ* FMCs, offering the potential to develop innovative methods for exploring biogeochemical cycles and controlling soil pollution.

In this study, we investigated active PAH-degrading bacteria in petroleum-contaminated soil using ^13^C-labelled phenanthrene (PHE) as a metabolic tracer, based on its widespread occurrence in nature and unique fused-ring angular structure [21, 22]. Through the innovative combination of RACS-SIP and genome-directed culture, we achieved the identification and cultivation of bacteria actively involved in PHE degradation within a real environment at the single-cell level. This study introduces the concept of *in situ* FMC and successfully utilizes these microbial cells to construct an *in situ* FMC, providing insights regarding FMC formation mechanisms. Our findings demonstrate the efficacy of our research method for the *in situ* identification, isolation, and cultivation of active PHE-degrading microorganisms at the single-cell level. These microbial entities have the potential to facilitate the construction of FMCs *in situ*. By performing single-cell genomic sequencing of sorted cells via RACS-SIP, we reconstructed the overall metabolic pathways used by active degraders and linked their identities to functions at the single-cell level, elucidating the mechanism of microbial community construction. Our study opens avenues for the exploration of specific and targeted organic pollutant-degrading microorganisms in diverse environments and offers valuable insights into the complex mechanisms underlying the construction of functional flora, expanding the available knowledge regarding their degradation processes.

## Materials and methods

### Sample collection

Soil contaminated with petroleum was obtained at depths ranging from 0 to 20 cm in Shengli Oil Field in China (37°52′ N, 118°56′ E). Upon arrival at the laboratory, a subset of soil samples was carefully preserved at −80°C to facilitate the initial DNA extraction process. The remaining soil samples were promptly stored at 4°C for subsequent RACS-SIP experiments. Subsequently, we analysed the petroleum content and specific soil characteristics of the samples, as outlined in Table S1.

### SIP microcosms and DNA ultracentrifugation

Microcosms were established in 150-ml serum bottles with 5 g of soil and 20 ml of phosphate-buffered mineral medium, in accordance with previous procedures [23]. Unlabelled PHE or ^13^C-labelled PHE (^13^C_14_-PHE) from Cambridge Isotope Laboratories, Inc. (USA) was added to achieve an initial concentration of 10 mg/L. Two biotic treatments were established, including soil with ^12^C-PHE and ^13^C-PHE, as well as a control involving unlabelled PHE-amended sterilized soils. Each treatment comprised six replicates incubated at 28°C with agitation (180 rpm) for 6 days. Soil samples were collected on Day 3 to avoid cross-feeding because the majority of PHE was degraded by Day 6 (>98%, Table S2).

**Figure 1:**
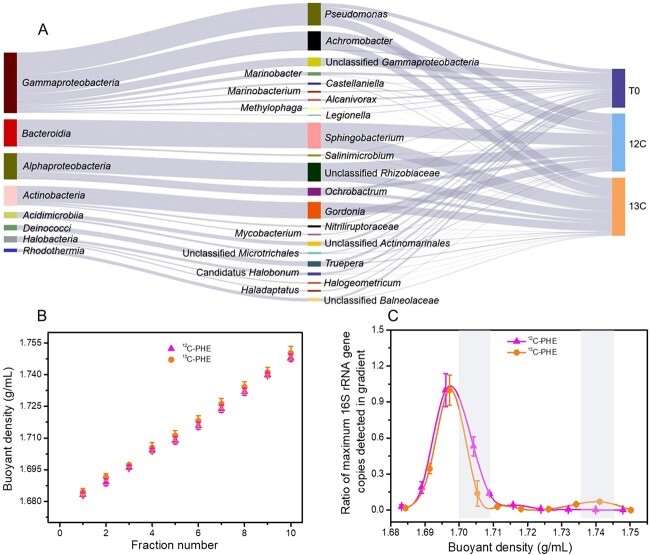
(A) Microbial community changes based on Sankey diagram. The selected taxa have a minimal relative abundance >1%. T0 represents microbial community in original soil sample. The selected taxa have a minimal relative abundance >1%. 12C and 13C represent microcosms with ^12^C-PHE and ^13^C-PHE, respectively, after 3 days of incubation. (B) Correlation between fraction number and buoyant density (g/ml) from DNA extracted from water samples in ^12^C_PHE and ^13^C_PHE treatments after 3 days of incubation. (C) Correlation between 16S rRNA gene abundance and buoyant density (BD, g/ml) in DNA extracted from the ^12^C_PHE and ^13^C_PHE microcosms. The “heavy” DNA fraction is highlighted.

DNA was extracted from each sample using the PowerSoil DNA Isolation Kit. DNA from the ^12^C_PHE and ^13^C_PHE microcosms was subjected to CsCl gradient ultracentrifugation through the mixing of ~5-μg DNA with Tris-EDTA/CsCl solution at a buoyant density (BD) of ~1.77 g/ml. Ultracentrifugation was performed in a Beckman Coulter L-100XP ultracentrifuge at 47 500 rpm for 48 h at 20°C. Subsequently, 14 fractions were collected from each tube to determine the relationship between BD and fraction number (Fig. 1B); DNA samples were purified through ethanol precipitation with glycogen assistance for quantification and amplicon sequencing [24, 25]. No sequences related to *Acinetobacter lwoffii* were detected in the SIP-stratified samples, indicating that the glycogen used was free of DNA contamination [26]. Details of this process are provided in the Supporting Information.

### Real-time quantitative polymerase chain reaction, 16S rRNA gene amplicon sequencing, and analysis

16S rRNA genes from DNA fractions of the ^12^C_PHE and ^13^C_PHE microcosms were amplified using the 515F/806R primer set [7, 27]. After gel purification, the quantitative polymerase chain reaction (qPCR) products were cloned into pGEM-T plasmids and sequenced. A standard curve was constructed using 10-fold serial dilutions of recombinant *Escherichia coli* sequences. The qPCR thermocycler protocol included 40 cycles with specific conditions, and DNA samples were amplified in triplicate. The ‘light’ and ‘heavy’ DNA fractions were identified based on the relationship between BD values and 16S rRNA gene abundance in SIP microcosms. Specifically, DNA fractions with BD values of 1.7042–1.7089 and 1.7342–1.7411 g/ml were categorized as light and heavy DNA fractions, respectively (Fig. 1C). For amplicon sequencing, the V4 region was amplified using the 515F/806R primer set [28]. Sequencing was performed on a MiSeq System (Illumina, USA) in a standard 2 × 250-bp paired-end pipeline. Paired-end 16S rRNA reads were merged using FLASH v1.2.11. Low-quality reads (length < 200 bp, > 2 ‘N’ bases, average quality <30) were filtered using a custom Python script. Subsequently, sequences were processed and analysed using Quantitative Insights into Microbial Ecology (QIIME2, pipeline v2019.10.0) [29]. DADA2 was utilized to obtain amplified sequence variants (ASVs) [30]. Taxonomic assignment of 16S rRNA gene sequences was performed with the SILVA database (release_138) [31].

Active PHE degraders in SIP microcosms were determined by comparison of relative enrichment factors (REFs), obtained using Eq. (1) from prior studies [32].


(1) \begin{equation*} REF=\left(\frac{A13\_ heavy}{A13\_ light}\right)\Big/\left(\frac{A12\_ heavy}{A12\_ light}\right) \end{equation*}

A13_heavy and A13_light denote ASV abundances in ^13^C-PHE treatments based on heavy and light DNA fractions, respectively, whereas A12_heavy and A12_light represent the same fractions from ^12^C-PHE treatments. Here, ASVs with REF > 2.0 among the top 100 ASVs were selected as active PHE degraders and subjected to phylogenetic analysis, as previously described [7, 21].

### Identification and isolation of ^13^C cells through RACS and single-cell genomic sequencing

To identify active PHE-degrading bacterial cells, samples from ^13^C_PHE microcosms were used for Raman-activated cell sorting (RACS); the ^12^C_PHE microcosms served as controls. Bacterial cells from *t* = 0 days were used for an RACS benchmarking experiment. The specific steps of this process were described in our previous work [12]. Briefly, samples were sonicated, centrifuged, and washed to prepare microbial cell pellets, which were spotted onto a sorting chip and subjected to Raman spectral acquisition with a 532-nm laser [33]. Spectral data were preprocessed for baseline correction and vector normalization using LabSpec 6 software [34]. Bacterial cells utilizing isotopically labelled substrates show redshift of the Raman band for phenylalanine. The position of this band in unlabelled cells is 1001–1003 cm^−1^. For functional cells that have incorporated ^13^C, this band exhibits a considerable redshift of −37 cm^−1^ [13, 35]. Thus, the positions of Raman bands detected via single-cell Raman spectroscopy (SCRS) from functional bacterial cells were analysed to establish the relationship between the observed redshift and ^13^C assimilation in the ^13^C_PHE microcosms. Details of this process are provided in the Supporting Information.

Single cells exhibiting a ^13^C shift were sorted using the Precision Single Cell Sorter (PRECI SCS) technique [34]. After active bacterial cells had been identified, those cells were individually sorted into cell receivers (Hooke Instruments Ltd., Changchun, China) loaded with cell lysis buffer. In total, 60 microbial cells were isolated, lysed, and subjected to Raman band identification and ^13^C-shift analysis. The entire process took less than 1 h to minimize changes in biological traits.

Genomic DNA from sorted cells was amplified through multiple displacement amplification (MDA) [16], verified by PCR using the primer set 27F/1492R, and sequenced using the PE150 strategy on the HiSeq X-ten platform (Illumina) [34]. Two gigabytes of sequencing data were acquired and subjected to filtering; reads with <80 nucleotides (nt) and mean quality scores <25 were removed. The remaining short sequences were assembled into contigs using the MEGAHIT assembler [36], resulting in contigs with lengths >1000 bp from the single-cell samples. After contig assembly, functional prediction and annotation were performed using Prokka v1.12.31 [37]. The genomes of the functional microbial cells were then assembled and output using MetaWRAP’s binning tools (v1.3.27) [38]. To investigate their functional genes and metabolic characteristics, Kyoto Encyclopedia of Genes and Genomes (KEGG) and AromaDeg databases were applied [9]. Functional gene sequences related to AromaDeg enzyme families were aligned using MAFFT software with the L-INS-i algorithm and specific parameters (E-value >0.00001, identity >50%, query coverage >50%, and topic coverage >30%). Taxonomic classification of the assembled bins was conducted with GTDB-Tk version 2.1.012 [39].

### Cultivation of active PHE degraders sorted via RACS

To cultivate active PHE degraders sorted by RACS from petroleum-contaminated soil, we aimed to adapt the conventional cultivation medium. Our approach involved utilizing metabolic and genetic insights obtained through genome binning of PHE degraders from single-cell genomic sequencing, which allows for the targeted cultivation of active microorganisms based on genomic analysis [17, 40]. We identified genes related to metabolism of vitamins (e.g. vitamins A, B1, B2, B3, B6, B7, B12, and lipoic acid) and trace metals essential to microbial growth (e.g. Ca, Cu, Zn, Co, Na, Mo, and Ni) within the assembled genome (Fig. 4). Moreover, the degraders exhibited genes encoding L-lactate dehydrogenase; resistance to aminoglycoside antibiotics was suggested by the presence of an aminoglycoside antibiotic-resistance gene. Consequently, we modified the conventional cultivation medium (Table S6) by supplementing it with 10 ml/L vitamin and mineral stock solutions, 5 mg/L streptomycin (an aminoglycoside antibiotic), and 50 mM of L-lactate to target the cultivation of active degraders from contaminated soil.

For the cultivation of active degraders, laser pulses were applied to transfer individual cells from a cell-coating chip to individual cell receivers containing 4 μl of modified minimal (MM) medium during the sorting process. In total, 60 single cells were sorted via RACS and rapidly transferred to MM medium. After 7 days of incubation at room temperature, culture solutions were spread on solid media and maintained under the same conditions for an additional 4 days to obtain pure bacterial isolates. Subsequently, genomic DNA was extracted, and the identities of the isolated organisms were determined through amplification of their 16S rRNA gene sequences using the universal bacterial primers 27F/1492R (Table S3), followed by sequencing.

### PHE degradation and construction of the *in situ* functional microbial consortium using RACS-sorted microorganisms

Two strains, designated JB-1 and JB-2, were successfully isolated and cultivated. PHE degradation experiments were conducted in 150-ml brown glass bottles containing 20-ml MM medium with a final PHE concentration of 50 mg/L; bacterial cell concentrations were adjusted to ~3 × 10^7^ colony-forming units (CFUs)/ml using the dilution plate counting method [11, 41]. Concurrently, to investigate the mechanism behind constructing a functional microbial flora for the *in situ* PHE degradation, we combined the two microorganisms (JB-1 and JB-2) at specific ratios that were determined according to their abundance ratio in the SIP microcosms after 3 days of incubation (~1:1; Fig. 2C), in the RACS-sorted cells (~1:1; Fig. 3D) and ^13^C-DNA fractions (~2:1; Fig. S2). Ultimately, suspensions of strains JB-1 and JB-2 were added to the bottles as monocultures or cocultures (CFU, 1:1, 1:2, or 2:1); noninoculated treatments served as blank controls. All tests were conducted in triplicate, using the same standards and incubation conditions implemented in the SIP microcosms. Samples were collected on Days 0, 3, and 6 for destructive sampling to extract PHE.

**Figure 2:**
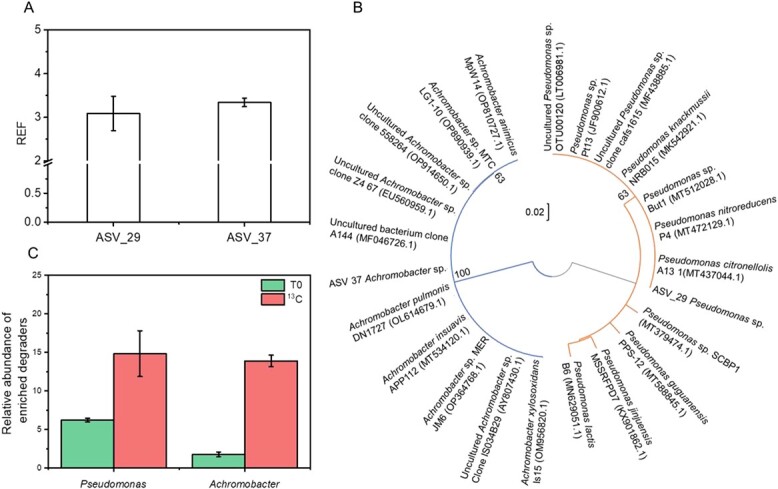
(A) The enrichment factor (REF) of ASVs from the SIP treatments. (B) Phylogenetic tree of SIP-identified ASVs based on their 16S rRNA gene sequences. Bootstrap values (expressed as percentages of 1200 replications) > 50% are shown at the branch points. Bar: 0.02 substitutions per nucleotide position. (C) The relative abundance of the enriched ASVs (*Achromobacter* and *Pseudomonas*) in the ^13^C_PHE microcosms after 3 days of incubation. T0 represents the original soil sample. Data are means ± standard deviation (*n* = 3).

**Figure 3:**
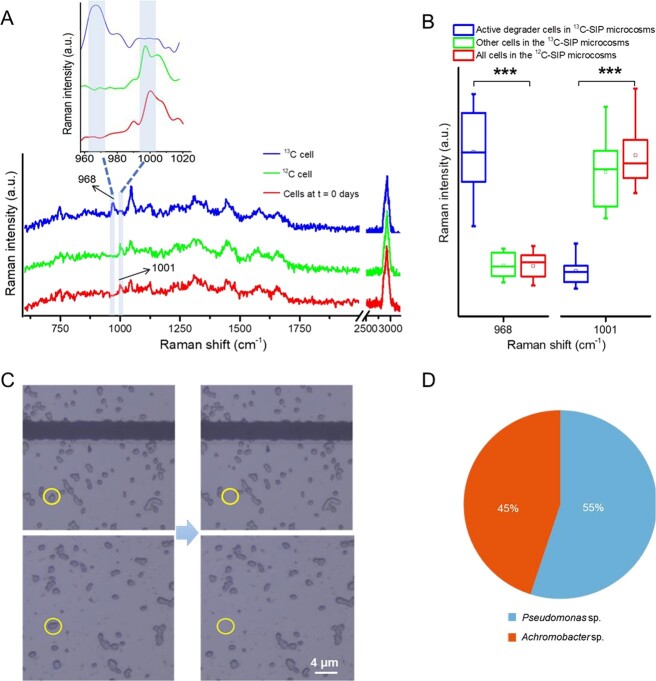
RACS of the active PHE degraders in soil. (A) Single-cell Raman spectra of cells after *in situ* incubation with ^12^C-PHE and ^13^C-PHE, tagged as ^12^C cell and ^13^C cell, respectively. Cells at *t* = 0 days indicate the Raman spectra of cells treated with ^13^C-PHE at time *t* = 0 days. Each spectrum represents an average of SCRS from detected cells (60 cells). (B) Intensities of Raman bands at 968 and 1001 cm^−1^ based on the active cells detected in the 13C-PHE microcosms, unlabelled cells in the ^13^C-PHE microcosms, and cells in the ^12^C-PHE microcosms. Comparisons denoted by asterisks (^*^^*^^*^) and a black bar are significantly different (one-way ANOVA, *P* < .001). (C) Active bacterial cells on the sorting chip were identified by SCRS from the ^13^C-PHE microcosms (left), and cells were ejected off the sorting chip by RACS (right). (D) The relative abundance of two RACS-sorted microorganisms based on the PE150 strategy of the Illumina HiSeq X-ten.

**Figure 4:**
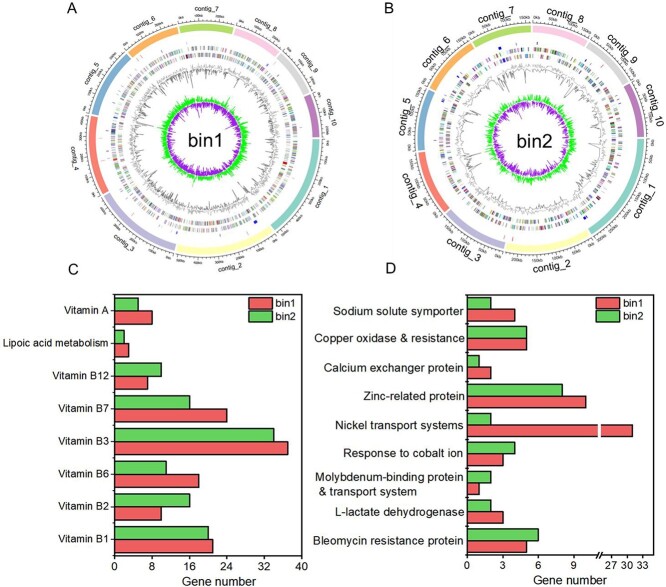
Circular representations of *Achromobacter* sp. (bin1; A) and *pseudomonas* sp. (bin2; B) from the sorted cells. From inside out: GC skew, GC percent, peaks out/inside the circle indicate values higher or lower than the average G + C content, regions in minus and plus strands (coloured by Cluster of Orthologous Group (COG) functional categories), and genes annotated by the CAZy database, as well as the Aromadag database. Contigs are highlighted in different colours in the outermost circle. (C) Counts of genes involved in the metabolism of cofactors, vitamins in the genomes of *Achromobacter* sp. (bin1) and *Pseudomonas* sp. (bin2). (D) Counts of genes associated with aminoglycoside antibiotic resistance, L-lactate and metal transport, oxidase, resistance, and exchanger in the assembled genomes.

### Isolation of highly efficient PHE-degrading bacteria that do not actively participate in PHE degradation and the construction of a functional microbial consortium

For comparison of bioremediation efficacy with the *in situ* FMC described above, we employed conventional isolation techniques to procure two proficient PHE-degrading strains, designated Isolate1 and Isolate2 (Fig. S5). Simultaneously, we generated a functional microbial consortium comparable to the consortium described above. Details of this section are provided in the Supporting Information. Although we identified >10 proficient PHE-degrading microorganisms, including Isolate1 and Isolate2, those organisms did not actively contribute to PHE degradation in oil-contaminated soil (Table S4).

### Bioremediation potential of two functional bacterial consortia in oil-contaminated soil

Considering that the two strains obtained from the functional bacterial consortia described above exhibited their highest PHE degradation efficiency when introduced at a CFU ratio of 1:1, we selected this ratio to investigate the functionality of the constructed FMC in natural soil. Microcosms were established within 150=ml serum bottles using the same procedure implemented for SIP incubations. In total, eight treatments were constructed: a sterile control (ST) utilizing soil sterilized with gamma-ray irradiation; a control with no added strains (CK); and treatments with only strain JB-1 (JB-1), only strain JB-2 (JB-2), a combination of strains JB-1 and JB-2 at a 1:1 CFU ratio (JB-1:JB-2), only Isolate1 (Isolate1), only Isolate2 (Isolate2), and a joint application of Isolate1 and Isolate2 at a 1:1 CFU ratio (Isolate1:Isolate2). Each treatment was performed in triplicate, and chemical analysis was conducted after 3-day incubation, as detailed in the following section.

### Chemical analysis

PHE in soil and MM medium was extracted at each sampling point (Day 0, 3, and 6), followed by analysis using gas chromatography–mass spectrometry (Agilent 7890), as previously described [42, 43]. Briefly, samples were fortified with recovery standards and extracted twice using dichloromethane. Subsequently, the organic extract was purified through a silica gel/alumina column and ultimately concentrated to ~0.5 ml. Prior to instrumental analyses, 1000 ng of hexamethylbenzene was introduced into the organic solvents as an internal standard.

### Statistical analysis

Data are presented as means ± standard deviations. Statistical analyses were conducted using Analysis of Variance (ANOVA) with SPSS version 24.0 and Origin 8.0 software. Clustering was accomplished utilizing the neighbour-joining method, and the phylogenetic tree was constructed via bootstrap analysis with 1200 iterations. Statistical significance was indicated by *P* values less than 0.05 (*P* < .05) or 0.001 (*P* < .001). Phylogenetic information for the isolated strain and active degrading bacteria was determined using BLAST and MEGA version 5.0 after multiple alignments.

## Results

### Microbial community dynamics and active PHE degrader identification via SIP

PHE degradation efficiency in biotic treatments reached 72–76% after 3 days of incubation, in contrast to 9–11% in sterile controls, confirming active PHE biodegradation (Table S2). The microbial community structure was relatively unchanged between the ^12^C_PHE and ^13^C_PHE treatments. However, a substantial shift was observed in soil microbial community structure between the original soil and treated soils (Fig. 1 & S1). In the original soil, the dominant bacteria (>5%) included unclassified *Gammaproteobacteria* (12.5%) and *Pseudomonas* (6.21%). After 3 days of incubation, the relative abundance of unclassified *Gammaproteobacteria* decreased to 0.25%, whereas *Pseudomonas* abundance significantly increased to 14.5%. Strains of *Achromobacter* (14.4%), unclassified *Rhizobiaceae* (14.5%), *Sphingobacterium* (19.9%), and *Gordonia* (12.7%) became the dominant microorganisms, although they were present at low levels in the original soil (0.32–1.77%).

Active degraders involved in ^13^C-PHE degradation were identified based on REF values. Calculations revealed that microorganisms represented by ASV_29 and ASV_37 exhibited high REF values of 3.09 and 3.34, respectively (Fig. 2). Phylogenetic analysis assigned these two ASVs to the genera *Pseudomonas* and *Achromobacter* (accession numbers: PP536048-PP536049); they exhibited the highest identities with *Pseudomonas* sp. A131 (MT437044.1) and *Achromobacter* sp. DN1727 (OL614679.1), respectively (Fig. 2C).

### *In situ* identification and sorting of PHE-degrading bacterial cells via RACS

In the ^13^C-PHE microcosms, SCRS analysis of 800 bacterial cells identified 60 cells with distinctive Raman bands, indicating ^13^C incorporation. These shifts were absent from the ^12^C-PHE microcosms (Fig. 3A), where all cells exhibited a common Raman band at 1001 cm^−1^ (a biomarker for unlabelled cells representing phenylalanine). Active degraders in the ^13^C-PHE microcosms displayed Raman shifts from 1001 to 968 cm^−1^, whereas no ^13^C-related shifts were observed in the ^12^C-PHE treatments. Raman intensity analysis revealed significantly higher intensities at 968 cm^−1^ but lower intensities at 1001 cm^−1^ (*P* < .001) for the active degrading cells in the ^13^C-PHE microcosms (Fig. 3B). Consequently, RACS enabled the sorting of single cells with identical ^13^C-shifted bands (Fig. 3C). After amplification, MDA products underwent single-cell genome sequencing, yielding two assembled genomes with completeness levels of 70.6% (bin1) and 76.1% (bin2) (Fig. 4 & Table S5). These genomes were affiliated with the genera *Achromobacter* and *Pseudomonas*; they were closely related to *Achromobacter* sp. DN1727 (OL614679.1) and *Pseudomonas* sp. A131 (MT437044.1), corresponding to SIP-identified ASV_37 and ASV_29, respectively. Additionally, whole-genome sequence comparison revealed elevated average nucleotide identity (ANI) values: 98.8% for bin1 and *Achromobacter pulmonis*, and 97.4% for bin2 and *Pseudomonas citronellolis*, both of which surpassed the intra- and inter-species threshold of 95% (Fig. S3). These results confirmed that bin1 and ASV_37 belong to the same species, as do bin2 and ASV_29, validating the identification of active degrading microorganisms obtained through SIP (Fig. 2).

### Metabolic characteristics and cultivation of active PHE degraders sorted via RACS

The assembled genomes were utilized to analyse the metabolic properties of sorted cells, offering insights into their cultivation potential. Genes associated with cofactor, vitamin, and carbohydrate metabolism were screened; genes related to metal transport, oxidase activity, resistance mechanisms, and ion exchange (essential to microbial growth) were also screened. The results revealed genes associated with the metabolism of various vitamins, including vitamins A, B1, B2, B3, B6, B7, B12, and lipoic acid (Fig. 4), as well as genes linked to trace metals vital for microbial growth, such as Ca, Cu, Zn, Co, Na, Mo, and Ni [44]. Additionally, the assembled genomes contained aminoglycoside antibiotic resistance proteins and L-lactate dehydrogenase (Fig. 4). These metabolic insights facilitated the development of a tailored MM medium for cultivation of active degraders obtained through RACS.

After cultivation, the culture solution was visibly turbid. After the cells had been transferred to MM agar plates, two target strains were successfully cultivated and purified from the sorted cells, designated *Pseudomonas* sp. JB-1 and *Achromobacter* sp. JB-2. Based on 16S rRNA gene sequence analysis, JB-1 shared 100% identity with ASV_29, whereas JB-2 exhibited identical sequences with ASV_37. The neighbour-joining phylogenetic dendrogram (Fig. S4) categorized these strains within the genera *Pseudomonas* and *Achromobacter*, forming subclades with ASV_29 and 37, respectively.

### Evaluation of PHE degradation by RACS-sorted functional bacteria and their consortium *in situ*

The strains *Pseudomonas* sp. JB-1 and *Achromobacter* sp. JB-2 exhibited robust growth in MM medium, utilizing PHE as the sole carbon source. Within 6 days, they achieved PHE biodegradation rates of 75.6% and 70.7%, respectively, whereas only 12.1% of PHE was removed in the control treatment (Fig. S6). Furthermore, the consortium of these two microorganisms displayed improved PHE degradation efficacy, particularly when combined at a 1:1 CFU ratio, reaching a maximum degradation rate of 90.7%. To compare the bioremediation performance of the FMC *in situ*, we isolated two highly efficient non-RACS-SIP identified PHE-degrading bacteria, Isolate1 and Isolate2. After 6 days of incubation under identical conditions, their degradation rates reached 87.8% and 90.7%, significantly surpassing the performance of strains JB-1 and JB-2 obtained through RACS (*P* < .05). Additionally, the PHE degradation efficiency exhibited by microbial consortia consisting of Isolate1 and Isolate2 exceeded 89.4%, representing a significant improvement over the consortium of JB-1 and JB-2 formed at the same ratio (*P* < .05). An outstanding PHE degradation rate of 96.1% was achieved when Isolate1 and Isolate2 were introduced at a 1:1 CFU ratio (Fig. S6).

### PHE degradation by two bacterial consortia in soil

**Figure 5:**
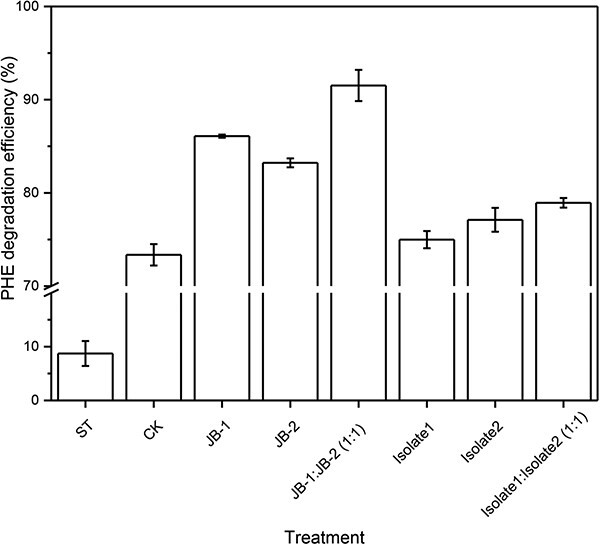
Soil PHE degradation efficiency of treatments adding different bacterial strains, including RACS-sorted microorganisms and the highly efficient degrading bacteria that do not actively participate in PHE degradation, as well as their constructed microbial consortia after 3 days of incubation. Data are means ± standard deviation; *n* = 3.

The introduced microorganisms and constructed FMCs exhibited considerable disparities in PHE degradation compared with the results in MM medium. Isolate1 failed to significantly enhance PHE degradation, displaying efficiency similar to microcosms without added microorganisms (*P* > .05) after 3 days of culture. Similarly, the addition of Isolate2 or simultaneous addition of Isolate1 and Isolate2 did not significantly improve PHE degradation, despite their effectiveness in MM medium (Fig. 5). However, the addition of JB-1 or JB-2 to soil significantly increased PHE degradation efficiency (83.2%–86.15%; *P* < .05) compared with Isolate1 or Isolate2 treatments (74.9%–77.1%), demonstrating their substantial contribution to PHE degradation in contaminated soil. The microbial consortium composed of JB-1 and JB-2 exhibited superior degradation (91.5%) compared with that of Isolate1 and Isolate2 (78.9%). Thus, the concurrent addition of JB-1 and JB-2 produced the highest PHE degradation efficiency, surpassing JB-1 or JB-2 alone, as well as the treatments containing Isolate1 or/and Isolate2 (Fig. 5). This finding contrasts with their performance in MM medium and highlights the pivotal role of RACS-sorted microorganisms in accelerating PHE degradation in real soil environments.

### PHE metabolic pathways of two RACS-sorted microorganisms in soil

**Figure 6:**
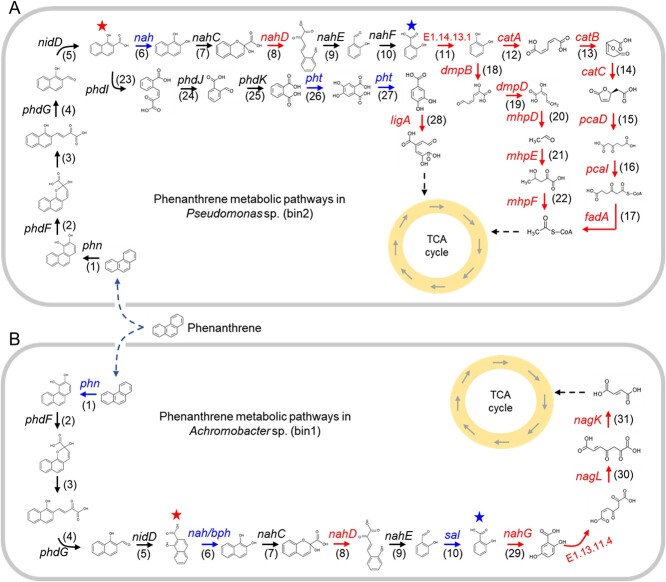
Reconstruction of PHE metabolic pathway of the sorted cells incorporating ^13^C-PHE characterized by SIP-RACS. The genes of *nidD*, *phdF-G*, *phd I-K*, *nahC* and *nahE* were not detected in both GhostKOALA annotation and AromaDeg database. The numbers stand for the enzymes as following: (1) phenanthrene dioxygenase (*phn*), (2) extradiol dioxygenase (*phdF*), (3) epimerase, (4) hydratase-aldolase (*phdG*), (5) aldehyde dehydrogenase (*nidD*), (6) naphthalene dioxygenase (*nah*), (7) 1,2-dihydroxynaphthalene dioxygenase *(nahC*), (8) 2-hydroxychromene-2-carboxylate isomerase (*nahD*), (9) trans-o-hydroxybenzylidenepyruvate hydratase-aldolase (*nahE*), (10) salicylaldehyde dehydrogenase (*nahF/Sal*), (11) salicylate 5-hydroxylase large subunit; (12) catechol 1,2-dioxygenase (*catA*), (13) muconate cycloisomerase (*catB*), (14) muconolactone D-isomerase (*catC*), (15) 3-oxoadipate enol-lactonase (*pcaD*), (16) 3-oxoadipate CoA-transferase, alpha subunit (*pcaI*), (17) acetyl-CoA acyltransferase (*fadA*), (18) catechol 2,3-dioxygenase (*dmpB*), (19) 2-hydroxymuconate-semialdehyde hydrolase (*dmpD*), (20) 2-keto-4-pentenoate hydratase (*mhpD*), (21) 4-hydroxy 2-oxovalerate aldolase (*mhpE*), (22) acetaldehyde dehydrogenase (*mhpF*), (23) 1-hydroxy-2-naphthoate dioxygenase (*phdI*), (24) 4-(2-carboxyphenyl)-2-oxobut-3-enoate aldolase (*phdJ*), (25) 2-formylbenzoate dehydrogenase (*phdK*), (26) phthalate 4,5-dioxygenase (*pht*), (27) 4,5-dihydroxyphthalate decarboxylase (*pht*), (28) protocatechuate 4,5-dioxygenase, alpha chain (*ligA*), (29) salicylate 5-hydroxylase large subunit (*nahG*), (30) maleylpyruvate isomerase (*nagL*), and (31) fumarylpyruvate hydrolase (*nagK*).

To elucidate the PHE biodegradation mechanisms detected through RACS-SIP and their roles in constructing the functional microbial community, we analysed PHE metabolic pathways in functional microorganisms sorted via RACS. We identified functional categories linked to xenobiotic biodegradation and metabolism, including the biodegradation of aromatic compounds (e.g. aminobenzoate, benzoate, bisphenol, and PAHs) within the sorted PHE-degrading bacterial cells (Fig. S7). Furthermore, we obtained the genomes (bin1 and bin2) of two RACS-sorted microorganisms. In *Achromobacter* sp. (bin1), we detected functional genes associated with the naphthalene/salicylate pathway of PHE degradation, including 2-hydroxychromene-2-carboxylate isomerase (*nahD*), salicylate hydroxylase (*nahG*), maleylpyruvate isomerase (*nagL*), and fumarylpyruvate hydrolase (*nagK*), via GhostKOALA annotation (Fig. 6). Furthermore, the AromaDeg database facilitated the identification of functional genes linked to naphthalene dioxygenase (*nah*) and salicylaldehyde dehydrogenase (*sal*) within bin1.

*Pseudomonas* sp. bin2 exhibited a distinct PHE metabolism pathway compared with bin1, and it showed the capacity to degrade PHE through multiple potential pathways (Fig. 6). These PHE degradation processes included the phthalate pathway, which is facilitated by the presence of functional genes such as phthalate 4,5-dioxygenase (*pht*), 4,5-dihydroxyphthalate decarboxylase (*pht*), and protocatechuate 4,5-dioxygenase (*ligA*). Additionally, bin2 can metabolize PHE through the naphthalene/salicylate pathway, although in a manner distinct from bin1. When converting PHE into catechol, bin2 utilizes enzymes including salicylate 5-hydroxylase large subunit, catechol 1,2-dioxygenase (*catA*), muconate cycloisomerase (*catB*), muconolactone D-isomerase (*catC*), 3-oxoadipate enol-lactonase (*pcaD*), 3-oxoadipate CoA-transferase (*pcaI*), and acetyl-CoA acyltransferase (*fadA*); alternatively, it uses catechol 2,3-dioxygenase (*dmpB*), 2-hydroxymuconate-semialdehyde hydrolase (*dmpD*), 2-keto-4-pentenoate hydratase (*mhpD*), 4-hydroxy 2-oxovalerate aldolase (*mhpE*), and acetaldehyde dehydrogenase (*mhpF*). Similar to bin1, specific genes such as *phn*, *nah*, and *pht* were exclusively identified using the AromaDeg database. Despite the distinct metabolic pathways utilized by the two bacteria, a fragment from the *phn* to *nahF/sal* gene was present in both genomes. This observation suggests that some metabolites within this pathway could be used by both microorganisms, alleviating their individual metabolic burdens.

By integrating data from both databases, we directly correlated the functions of two active PHE degraders with their genotypes, successfully mapping their PHE metabolism pathways in a relatively comprehensive manner and thus reinforcing their role in PHE degradation (Fig. 6). This analysis underscored the divergence in metabolic PHE processes among functional microbial flora *in situ*. Our findings identified diverse metabolic pathways used by various microorganisms within the functional microbial community, highlighting their potential contribution to effective microbial community construction for efficient targeted pollutant degradation.

## Discussion

Soil, a pivotal ecosystem, plays crucial roles in global climate change, element cycling, and pollutant transformation [45]. Soil microbial consortia, comprising diverse and complex microbial communities, pose inherent challenges for cultivation. Cutting-edge approaches for studies of specific soil functional flora include SIP and its integrated methodologies, such as magnetic-nanoparticle mediated isolation (MMI)-SIP and sequencing, which enable comprehensive exploration of metabolic mechanisms [7, 9, 42]. Yet, SIP and MMI-SIP investigations of environmental functional consortia face substantial challenges related to the availability of comprehensive genome data for specific microorganisms, hindering the linkage of these communities to functional genes and pathways, as well as the isolation and culture individual microorganisms for *in situ* FMC construction [12]. To overcome these challenges, we integrated SIP, RACS, and single-cell genome sequencing approaches. Through single-cell Raman analysis of soil microbial consortia fed ^13^C-PHE, we accurately identified active bacterial cells with PHE-metabolizing capabilities. Single-cell genome sequencing achieved >70% genome coverage, facilitating comprehensive exploration of functional genes associated with PHE metabolic pathways. Comparison of our findings with lab-cultivated PHE-degrading bacteria that were not detected through RACS-SIP highlighted differences in soil microbial compositions for *in situ* PHE metabolism, underscoring the limitations of pure culture-based methods. Thus, *in situ*, single-cell precision identification and isolation are vital for comprehensive analysis of FMCs and resource discovery.

Here, we successfully cultivated microorganisms capable of *in situ* PHE degradation in soil using RACS-SIP and genome-guided direct cultivation, and proposed the innovative concept of *in situ* FMC construction, which can effectively contribute to pollutant removal in real environments. In the context of FMC construction, researchers have explored two distinct strategies: bottom–up and top–down approaches, which provide diverse insights into the establishment of FMCs [19, 46]. Bottom–up approaches involve reconstructing metabolic networks based on the genomes of microbiome members and utilizing network analysis tools to design microbial consortia with specific functions [19]. This method is the main focus of synthetic biologists, and it centres on the development of tools for synthetic microbial communities to enable investigation of community-targeted functions [19, 47–49]. Conversely, the top–down strategy is primarily utilized by microbiologists and omics scientists. In this strategy, functional consortia are obtained through domestication under controlled conditions. The manipulation of physical and chemical environmental conditions guides the ecological selection of existing microorganisms from complex microbial communities, allowing researchers to screen for functional members and construct FMCs [19]. Compared with the methods outlined above, our proposed *in situ* FMC construction technique is centred on the functional microorganisms that are actually engaged in pollutant degradation in real environments. Precise, controlled addition of specific functional microorganisms—according to their natural community proportions—enabled us to construct an artificial *in situ* FMC. This consortium accurately mirrors the composition of the native functional microbial community in the soil that is actively engaged in pollutant degradation, thereby enhancing the efficacy of pollutant removal. Although our method is promising, some challenges and limitations persist, especially regarding genome integrity. Improvements concerning genome integrity are essential in our approach to studies of functional microbial cells; such improvements will streamline the acquisition of comprehensive genetic and metabolic data, extensively influencing analyses of the pollutant metabolic characteristics of these microorganisms. Moreover, this improvement will greatly advance the development of customized culture media.

Although our *in situ* FMC had lower degradation efficiency under MM medium conditions than conventionally designed functional groups, it displayed superior PHE remediation effects in real soil. Simultaneously, although microorganisms isolated through RACS-SIP exhibited lower degradation capacity on culture medium compared with conventionally isolated strains, they demonstrated significantly greater PHE removal capacities in native soil (*P* < .05). Previous research has highlighted the obstacles encountered by many microorganisms, particularly non-native functional strains, in achieving effective remediation when introduced into real-world environments [3, 50, 51]. These challenges are related to the intricate nature of the *in situ* milieu, including competition with indigenous microbial populations, limited nutrient availability, and the presence of inhibitory compounds [41, 51]. Despite the recognition of such underlying factors, comprehensive exploration from the perspective of functional microbial communities participating in *in situ* degradation has remained elusive. Our study fills this void by examining functional microbiomes *in situ*. By utilizing RACS-SIP and genome-guided direct cultivation, we successfully identified functional microorganisms capable of pollutant degradation *in situ* and facilitated their proliferation. This pattern suggests that microorganisms obtained through conventional cultivation methods possess pollutant tolerance but lack genuine *in situ* degradation capabilities. Our findings highlight the potential of artificially constructed functional microbial populations based on comprehensive understanding of *in situ* functional microorganisms, which exhibit promises of sustained and efficient pollutant degradation.

The establishment of an *in situ* FMC hinges on acquiring functional microorganisms, which can be particularly challenging due to the difficulty of detecting and culturing such microorganisms. Although we previously utilized RACS-SIP to pinpoint active PHE degraders and analyse their degradation mechanisms in wastewater, their cultivation has proven elusive [12]. To overcome this limitation, we integrated RACS-SIP with genome-guided cultivation, successfully cultivating targeted functional microorganisms fo*r in situ* soil PHE metabolism. In this study, we supplemented the culture medium with antibiotics to prevent contamination by other microorganisms; we included essential vitamins, minerals, and a readily utilized carbon source (L-lactic acid) to support microbial growth. Our approach demonstrates the efficacy of coupling RACS-SIP with genome-guided cultivation to identify and cultivate specific functional microorganisms within complex soil microbial communities, outlining a promising approach for investigating pollutant metabolism and element recycling in natural habitats.

Based on the genome sequences of functional bacterial cells, we clarified the metabolic mechanisms underlying the FMC. *Achromobacter* sp. displayed ANI > 95% with *A. pulmonis*, suggesting that it is a subspecies of *A. pulmonis*. Although *A. pulmonis* is a reported degrader of bispyribac sodium [52, 53], its role in the degradation of persistent organic pollutants (e.g. PAHs) was previously unreported. Here, we discovered the PHE degradation capability of this organism, reconstructed its comprehensive PHE metabolic pathway, and identified key genes involved in the *in situ* PHE biodegradation, including the catalytic naphthalene dioxygenase. For *Pseudomonas* sp., ANI indicated that it belongs to *P. citronellolis*, possibly as a distinct subspecies. Despite its presence in PAH-contaminated environments and its PAH degradation potential [54, 55], functional genes and metabolic pathways related to PHE degradation were previously unknown in this species. The present study utilized the KEGG and AromaDeg databases to reveal the PHE degradation mechanism of this strain *in situ* through single-cell genome sequencing. We reconstructed three nearly complete PHE metabolic pathways and identified the key enzymes in those pathways, including PHE/naphthalene dioxygenase. The isolated *Pseudomonas* microorganisms exhibited more diverse PHE metabolic pathways than *Achromobacter* sp., and distinct pathways were observed in these two strains. Based on the integrated PHE degradation efficiencies of these strains, our results suggests that a consortium consisting of two functional microorganisms with differing PHE metabolic pathways offers distinct advantages for enhancing PHE degradation efficiency compared with a single strain [19, 56]. These advantages include the division of labour and cooperation (including sharing metabolic resources) within the microbial community, which can decrease the amount of exogenous carrier PHE and alleviate the metabolic burden imposed by PHE on individual community members. Furthermore, because this microbial consortium participates in PHE degradation within its native soil environment, the exchange of information among community members and other soil microorganisms may contribute to overall consortium stability and efficacy during pollutant degradation [57]. Consequently, FMCs exhibiting diverse pollutant metabolic pathways may enhance the stability of PHE degradation. This study provides a single-cell-level approach to clarifying the molecular mechanisms involved in organic pollutant bioremediation by microbial consortia.

## Conclusions

We developed an innovative system for *in situ* functional microbial identification, sorting, cultivation, and consortium construction to investigate the *in situ* mechanisms of PHE degradation in an FMC. This system involved the precise identification, sorting, and cultivation of functional microorganisms at the single-cell level, resulting in the creation of an *in situ* FMC. Utilizing RACS-SIP, we successfully identified and sorted PHE-degrading bacterial cells belonging to *Achromobacter* sp. and *Pseudomonas* sp. and then obtained their cultures through genome-guided cultivation. Although conventional methods led to higher PHE degradation by functional flora under controlled culture conditions, our *in situ* FMCs exhibited significantly enhanced PHE degradation in real soil. Strains isolated via conventional methods, although more efficient in culture media, demonstrated less effectiveness when applied to natural soil. This finding suggests that conventionally isolated microorganisms possess high pollutant tolerance but lack the capacity to efficiently degrade pollutants in an *in situ* setting. Our findings underscore the potential of artificially constructed FMCs based on comprehensive investigation of *in situ* functional microorganisms. By inoculating these populations into natural soil, efficient pollutant degradation is attainable. Furthermore, we directly linked functional microorganisms to their functional genes and metabolic pathways through single-cell sequencing, thus enhancing the understanding of active metabolic mechanisms in the *in situ* FMC. Our technical system underscores the significance of *in situ*, single-cell precision identification, isolation, and cultivation of functional microorganisms, which are crucial to *in situ* microbial resource exploration and FMC construction. This study provides a valuable approach for investigating the metabolic mechanisms of *in situ* functional bacterial communities at the single-cell level, as well as ensuring their deliberate construction. Moreover, these findings have considerable relevance for the investigation of various FMCs, including those of archaea and fungi, engaged in elemental cycling and pollutant transformation within complex ecosystems.

## Supplementary Material

Supporting_information-data_wrae115

non-data_SIP_wrae115

## Data Availability

Sequence data are available at NCBI under the project accession PRJNA1091787.

## References

[1] Zhou ZC , Liu Y, Pan J et al. *Gammaproteobacteria* mediating utilization of methyl-, sulfur- and petroleum organic compounds in deep ocean hydrothermal plumes. ISME J*.* 2020;14:3136–48. 10.1038/s41396-020-00745-5 32820229 PMC7784996

[2] Whitman WB , Coleman DC, Wiebe WJ. Prokaryotes: the unseen majority. Proc Natl Acad Sci US*A* 1998;95:6578–83. 10.1073/pnas.95.12.6578 9618454 PMC33863

[3] Li J , Zhang D, Luo C et al. *In situ* discrimination and cultivation of active degraders in soils by genome-directed cultivation assisted by SIP-Raman-activated cell sorting. Environ Sci Techno*l* 2023;57:17087–98. 10.1021/acs.est.3c04247 37823365

[4] Yuan J , Zhao K, Tan X et al. Perspective on the development of synthetic microbial community (syncom) biosensors. Trends Biotechno*l* 2023;41:1227–36. 10.1016/j.tibtech.2023.04.007 37183053

[5] Moreno-Forero SK , van der Meer JR. Genome-wide analysis of *Sphingomonas wittichii* rw1 behaviour during inoculation and growth in contaminated sand. ISME *J* 2015;9:150–65. 10.1038/ismej.2014.101 24936762 PMC4274413

[6] Li J , Luo C, Song M et al. Biodegradation of phenanthrene in polycyclic aromatic hydrocarbon-contaminated wastewater revealed by coupling cultivation-dependent and -independent approaches. Environ Sci Techno*l* 2017;51:3391–401. 10.1021/acs.est.6b04366 28181806

[7] Li J , Luo C, Zhang G et al. Coupling magnetic-nanoparticle mediated isolation (MMI) and stable isotope probing (SIP) for identifying and isolating the active microbes involved in phenanthrene degradation in wastewater with higher resolution and accuracy. Water Re*s* 2018;144:226–34. 10.1016/j.watres.2018.07.036 30032019

[8] Li J , Zhang D, Song M et al. Novel bacteria capable of degrading phenanthrene in activated sludge revealed by stable-isotope probing coupled with high-throughput sequencing. Biodegradatio*n* 2017;28:423–36. 10.1007/s10532-017-9806-9 28956196

[9] Thomas F , Corre E, Cebron A. Stable isotope probing and metagenomics highlight the effect of plants on uncultured phenanthrene-degrading bacterial consortium in polluted soil. ISME J*.* 2019;13:1814–30. 10.1038/s41396-019-0394-z 30872807 PMC6775975

[10] Dumont MG , Murrell JC. Stable isotope probing - linking microbial identity to function. Nat Rev Microbiol*.* 2005;3:499–504. 10.1038/nrmicro1162 15886694

[11] Li J , Luo C, Zhang D et al. Autochthonous bioaugmentation modified bacterial diversity of phenanthrene degraders in pah-contaminated wastewater as revealed by DNA-stable isotope probing. Environ Sci Techno*l* 2018;52:2934–44. 10.1021/acs.est.7b05646 29378393

[12] Li J , Zhang D, Li B et al. Identifying the active phenanthrene degraders and characterizing their metabolic activities at the single-cell level by the combination of magnetic-nanoparticle-mediated isolation, stable-isotope probing, and Raman-activated cell sorting (MMI–SIP–RACS). Environ Sci Techno*l* 2022;56:2289–99. 10.1021/acs.est.1c04952 35061946

[13] Song YZ , Yin HB, Huang WE. Raman activated cell sorting. Curr Opin Chem Bio*l* 2016;33:1–8. 10.1016/j.cbpa.2016.04.002 27100046

[14] Li H-Z , Yang K, Liao H et al. Active antibiotic resistome in soils unraveled by single-cell isotope probing and targeted metagenomics. Proc Natl Acad Sci US*A* 2022;119:e2201473119. 10.1073/pnas.2201473119 36161886 PMC9546533

[15] Berry D , Mader E, Lee TK et al. Tracking heavy water (D_2_O) incorporation for identifying and sorting active microbial cells. Proc Natl Acad Sci US*A* 2015;112:E194–203. 10.1073/pnas.1420406112 25550518 PMC4299247

[16] Jing XY , Gou HL, Gong YH et al. Raman-activated cell sorting and metagenomic sequencing revealing carbon-fixing bacteria in the ocean. Environ Microbio*l* 2018;20:2241–55. 10.1111/1462-2920.14268 29727057 PMC6849569

[17] Carini P . A "cultural" renaissance: genomics breathes new life into an old craft. mSystem*s* 2019;4:5. 10.1128/mSystems.00092-19 PMC6533372 31219785

[18] Cross KL , Campbell JH, Balachandran M et al. Targeted isolation and cultivation of uncultivated bacteria by reverse genomics. Nat Biotechno*l* 2019;37:1314–21. 10.1038/s41587-019-0260-6 31570900 PMC6858544

[19] Cao Z , Yan W, Ding M et al. Construction of microbial consortia for microbial degradation of complex compounds. Front Bioeng Biotec*h* 2022;10:1051233. 10.3389/fbioe.2022.1051233 PMC9763274 36561050

[20] Wang X , Teng Y, Wang X et al. Nitrogen transfer and cross-feeding between *Azotobacter chroococcum* and *Paracoccus aminovorans* promotes pyrene degradation. ISME *J* 2023;17:2169–81. 10.1038/s41396-023-01522-w 37775536 PMC10689768

[21] Li J , Luo C, Zhang D et al. Stable-isotope probing enabled cultivation of the indigenous strain *Ralstonia* sp. M1 capable of degrading phenanthrene and biphenyl in industrial wastewater. Appl Environ Microbio*l* 2019;85:AEM.00511–9. 10.1128/aem.00511-19 PMC6606873 31053587

[22] Dai Y , Li J, Wang S et al. Unveiling the synergistic mechanism of autochthonous fungal bioaugmentation and ammonium nitrogen biostimulation for enhanced phenanthrene degradation in oil-contaminated soils. J Hazard Mate*r* 2024;465:133293. 10.1016/j.jhazmat.2023.133293 38141301

[23] Dai Y , Li J, Yang X et al. New insight into the mechanisms of autochthonous fungal bioaugmentation of phenanthrene in petroleum contaminated soil by stable isotope probing. J Hazard Mate*r* 2023;452:131271. 10.1016/j.jhazmat.2023.131271 36989785

[24] Coyotzi S , Pratscher J, Murrell JC et al. Targeted metagenomics of active microbial populations with stable-isotope probing. Curr Opin Biotechno*l* 2016;41:1–8. 10.1016/j.copbio.2016.02.017 26946369

[25] Neufeld JD , Vohra J, Dumont MG et al. DNA stable-isotope probing. Nat Proto*c* 2007;2:860–6. 10.1038/nprot.2007.109 17446886

[26] Bartram AK , Poon C, Neufeld JD. Nucleic acid contamination of glycogen used in nucleic acid precipitation and assessment of linear polyacrylamide as an alternative co-precipitant. BioTechnique*s* 2009;47:1019–22. 10.2144/000113276 20041853

[27] Zhao X , Li J, Zhang D et al. Unveiling the novel role of ryegrass rhizospheric metabolites in benzo[a]pyrene biodegradation. Environ In*t* 2023;180:108215. 10.1016/j.envint.2023.108215 37741005

[28] Li J , Luo C, Zhang D et al. Diversity of the active phenanthrene degraders in PAH-polluted soil is shaped by ryegrass rhizosphere and root exudates. Soil Biol Bioche*m* 2019;128:100–10. 10.1016/j.soilbio.2018.10.008

[29] Bolyen E , Rideout JR, Dillon MR et al. Reproducible, interactive, scalable and extensible microbiome data science using qiime 2. Nat Biotechno*l* 2019;37:852–7. 10.1038/s41587-019-0209-9 31341288 PMC7015180

[30] Callahan BJ , McMurdie PJ, Rosen MJ et al. Dada2: high-resolution sample inference from illumina amplicon data. Nat Method*s* 2016;13:581–3. 10.1038/nmeth.3869 27214047 PMC4927377

[31] Quast C , Pruesse E, Yilmaz P et al. The silva ribosomal RNA gene database project: improved data processing and web-based tools. Nucleic Acids Re*s* 2013;41:D590–6. 10.1093/nar/gks1219 23193283 PMC3531112

[32] Sun Y , Yin M, Zheng D et al. Different acetonitrile degraders and degrading genes between anaerobic ammonium oxidation and sequencing batch reactor as revealed by stable isotope probing and magnetic-nanoparticle mediated isolation. Sci Total Enviro*n* 2021;758:143588. 10.1016/j.scitotenv.2020.143588 33218816

[33] Cui L , Yang K, Li H-Z et al. Functional single-cell approach to probing nitrogen-fixing bacteria in soil communities by resonance Raman spectroscopy with N-15(2) labeling. Anal Che*m* 2018;90:5082–9. 10.1021/acs.analchem.7b05080 29557648

[34] Wang Y , Xu J, Kong L et al. Raman-activated sorting of antibiotic-resistant bacteria in human gut microbiota. Environ Microbio*l* 2020;22:2613–24. 10.1111/1462-2920.14962 32114713 PMC7383503

[35] Wang Y , Huang WE, Cui L et al. Single cell stable isotope probing in microbiology using Raman microspectroscopy. Curr Opin Biotechno*l* 2016;41:34–42. 10.1016/j.copbio.2016.04.018 27149160

[36] Li D , Liu C-M, Luo R et al. Megahit: an ultra-fast single-node solution for large and complex metagenomics assembly via succinct de Bruijn graph. Bioinformatic*s* 2015;31:1674–6. 10.1093/bioinformatics/btv033 25609793

[37] Seemann T . Prokka: rapid prokaryotic genome annotation. Bioinformatic*s* 2014;30:2068–9. 10.1093/bioinformatics/btu153 24642063

[38] Uritskiy GV , DiRuggiero J, Taylor J. Metawrap-a flexible pipeline for genome-resolved metagenomic data analysis. Microbiom*e* 2018;6:158. 10.1186/s40168-018-0541-1 30219103 PMC6138922

[39] Chaumeil PA , Mussig AJ, Hugenholtz P et al. Gtdb-tk v2: memory friendly classification with the genome taxonomy database. Bioinformatic*s* 2022;38:5315–6. 10.1093/bioinformatics/btac672 36218463 PMC9710552

[40] Droge J , McHardy AC. Taxonomic binning of metagenome samples generated by next-generation sequencing technologies. Brief Bioinfor*m* 2012;13:646–55. 10.1093/bib/bbs031 22851513

[41] Li J , Peng K, Zhang D et al. Autochthonous bioaugmentation with non-direct degraders: a new strategy to enhance wastewater bioremediation performance. Environ In*t* 2020;136:105473. 10.1016/j.envint.2020.105473 31999970

[42] Li J , Luo C, Zhang D et al. The catabolic pathways of in situ rhizosphere PAH degraders and the main factors driving PAH rhizoremediation in oil-contaminated soil. Environ Microbio*l* 2021;23:7042–55. 10.1111/1462-2920.15790 34587314

[43] Bao J , Li J, Jiang L et al. New insight into the mechanism underlying the effect of biochar on phenanthrene degradation in contaminated soil revealed through DNA-sip. J Hazard Mate*r* 2022;438:129466. 10.1016/j.jhazmat.2022.129466 35803194

[44] Lewis WH , Tahon G, Geesink P et al. Innovations to culturing the uncultured microbial majority. Nat Rev Microbio*l* 2021;19:225–40. 10.1038/s41579-020-00458-8 33093661

[45] Hartmann M , Six J. Soil structure and microbiome functions in agroecosystems. Nat Rev Earth En*v* 2023;4:4–18. 10.1038/s43017-022-00366-w

[46] Lawson CE , Harcombe WR, Hatzenpichler R et al. Common principles and best practices for engineering microbiomes. Nat Rev Microbio*l* 2019;17:725–41. 10.1038/s41579-019-0255-9 31548653 PMC8323346

[47] Mee MT , Collins JJ, Church GM et al. Syntrophic exchange in synthetic microbial communities. Proc Natl Acad Sci US*A* 2014;111:E2149–56. 10.1073/pnas.1405641111 24778240 PMC4034247

[48] Scott SR , Din MO, Bittihn P et al. A stabilized microbial ecosystem of self-limiting bacteria using synthetic quorum-regulated lysis. Nat Microbio*l* 2017;2:17083. 10.1038/nmicrobiol.2017.83 28604679 PMC5603288

[49] Glass DS , Riedel-Kruse IH. A synthetic bacterial cell-cell adhesion toolbox for programming multicellular morphologies and patterns. Cel*l* 2018;174:649–58.e16. 10.1016/j.cell.2018.06.041 30033369

[50] Nandy S , Andraskar J, Lanjewar K et al. Challenges in bioremediation: From lab to land. In: Saxena G, Kumar V, Shah MP (eds.), Bioremediation for Environmental Sustainabilit*y*. Elsevier, 561–83.

[51] Luo C , Zhao X, Zhang D et al. Toward a more comprehensive understanding of autochthonous bioaugmentation (aba): cases of aba for phenanthrene and biphenyl by *Ralstonia* sp. M1 in industrial wastewater. ACS ES&T Wate*r* 2021;1:1390–400. 10.1021/acsestwater.0c00257

[52] Vandamme P , Moore ERB, Cnockaert M et al. *Achromobacter animicus* sp. nov., *Achromobacter mucicolens* sp. nov., *Achromobacter pulmonis* sp. nov. and *Achromobacter spiritinus* sp. nov., from human clinical samples. Syst Appl Microbio*l* 2013;36:1–10. 10.1016/j.syapm.2012.10.003 23219252

[53] Ahmad F , Anwar S, Firdous S et al. Biodegradation of bispyribac sodium by a novel bacterial consortium bdam: optimization of degradation conditions using response surface methodology. J Hazard Mate*r* 2018;349:272–81. 10.1016/j.jhazmat.2017.12.065 29438823

[54] Oyehan TA , Al-Thukair AA. Isolation and characterization of PAH-degrading bacteria from the eastern province, Saudi Arabia. Mar Pollut Bul*l* 2017;115:39–46. 10.1016/j.marpolbul.2016.11.007 27912917

[55] Jacques RJS , Santos EC, Bento FM et al. Anthracene biodegradation by *pseudomonas* sp. isolated from a petrochemical sludge landfarming site. Int Biodeterior Biodegra*d* 2005;56:143–50. 10.1016/j.ibiod.2005.06.005

[56] McCarty NS , Ledesma-Amaro R. Synthetic biology tools to engineer microbial communities for biotechnology. Trends Biotechno*l* 2019;37:181–97. 10.1016/j.tibtech.2018.11.002 30497870 PMC6340809

[57] Stenuit B , Agathos SN. Deciphering microbial community robustness through synthetic ecology and molecular systems synecology. Curr Opin Biotechno*l* 2015;33:305–17. 10.1016/j.copbio.2015.03.012 25880923

