# Supplementary material for: Microbial consortium assembly and functional analysis via isotope labelling and single-cell manipulation of polycyclic aromatic hydrocarbon degraders
Source: ISME J. 2024 Jun 24;18(1):wrae115. doi: 10.1093/ismejo/wrae115 (PMC11256997; doi:10.1093/ismejo/wrae115)
Supplement: Supporting_information-data_wrae115 [file supporting_information-data_wrae115.pdf]

Supplementary Materials for

Microbial consortium assembly and functional analysis via isotope labelling and single-cell manipulation of polycyclic aromatic hydrocarbon degraders

Jibing Li[a,b], Chunling Luo[a,b,]*, Xixi Cai[c], Dayi Zhang[d,e], Guoqing Guan [a,b], Bei Li[f,g], Gan Zhang[a,b]

[a]State Key Laboratory of Organic Geochemistry and Guangdong-Hong Kong-Macao Joint Laboratory for Environmental Pollution and Control, Guangzhou Institute of Geochemistry, Chinese Academy of Sciences, Guangzhou 510640, China

[b]University of Chinese Academy of Sciences, Beijing 100039, China

[c]Guangdong Key Laboratory of Ornamental Plant Germplasm Innovation and Utilization, Environmental Horticulture Research Institute, Guangdong Academy of Agricultural Sciences, Guangzhou 510640, China

[d]Key Laboratory of Groundwater Resources and Environment, Ministry of Education, Jilin University, Changchun 130012, China

[e]College of New Energy and Environment, Jilin University, Changchun 130021, China

[f]State Key Lab of Applied Optics, Changchun Institute of Optics, Fine Mechanics and Physics, Chinese Academy of Sciences, 130033, Changchun, China

[g]HOOKE Instruments Ltd., 130033, Changchun, China

*Corresponding author: Dr. Chunling Luo

E-mail: clluo@gig.ac.cn Tel.: +86-20-85290290; Fax: +86-20-85290706

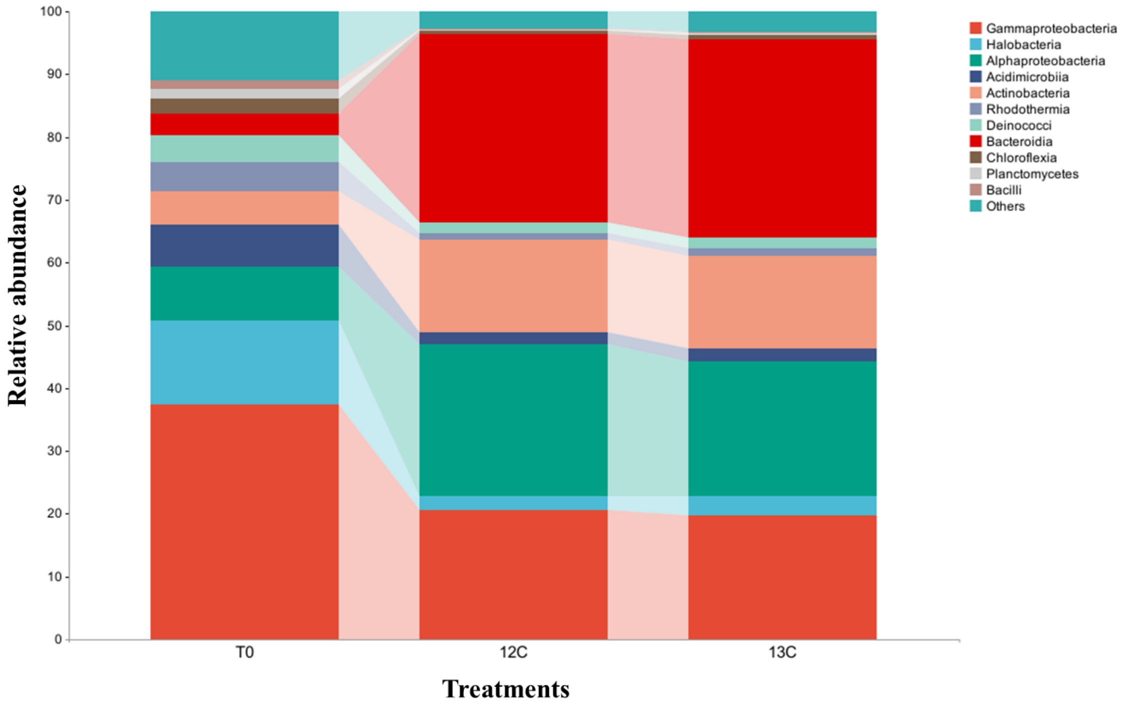

Figure S1. Relative abundance of 16S rRNA defined bacterial genera in the ^{12}C_PHE (12C) and ^{13}C_PHE (13C) microcosms. The selected taxa have a minimal relative abundance greater than 1%. T0 represents microbial community in original soil sample.

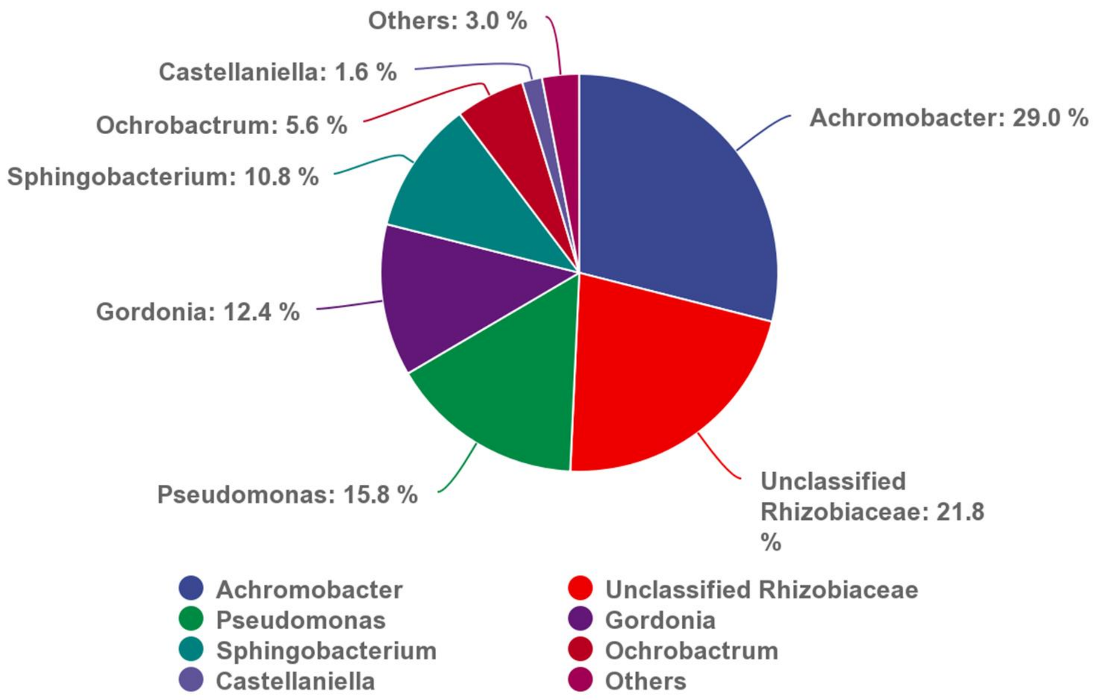

Figure S2. Microbial composition in the ^{13}C-DNA heavy fractions of the ^{13}C_PHE microcosms at the genus level. The selected taxa have a minimal relative abundance greater than 1%.

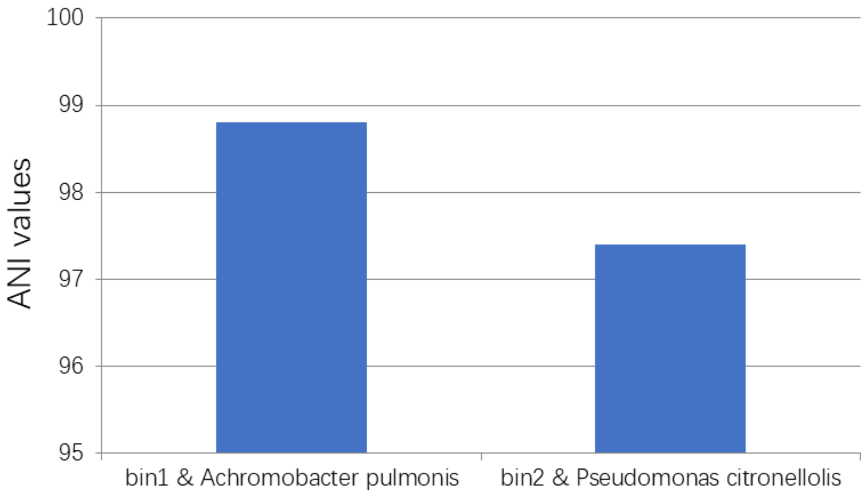

Figure S3. ANI values between bin1 & *Achromobacter pulmonis*, and bin2 & *Pseudomonas citronellolis*.

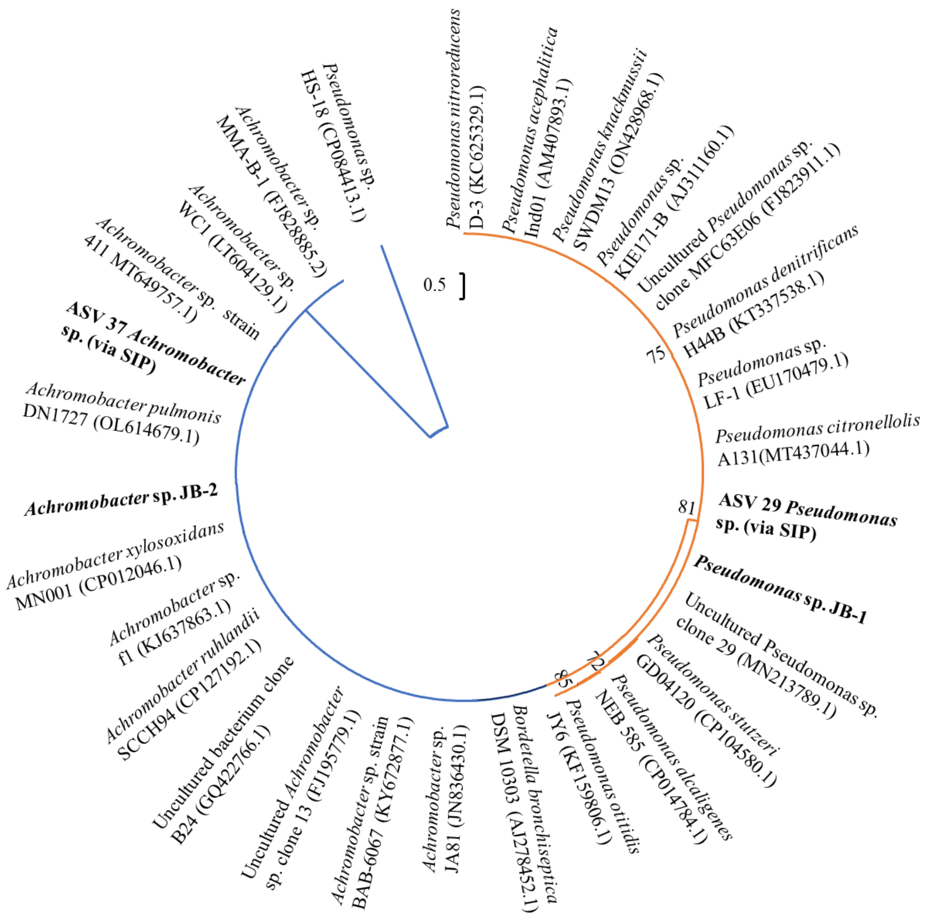

Figure 4. Phylogenetic tree of the RACS-isolated active PHE degraders (JB-1 and JB-2). Neighbor-joining tree based on 16S ribosomal RNA (rRNA) gene sequences showing the phylogenetic positions of these strains and representatives of other related taxa. Bootstrap values (expressed as percentages of 1200 replications) > 50% are shown at the branch points. The bar indicates 0.5 substitutions per nucleotide position.

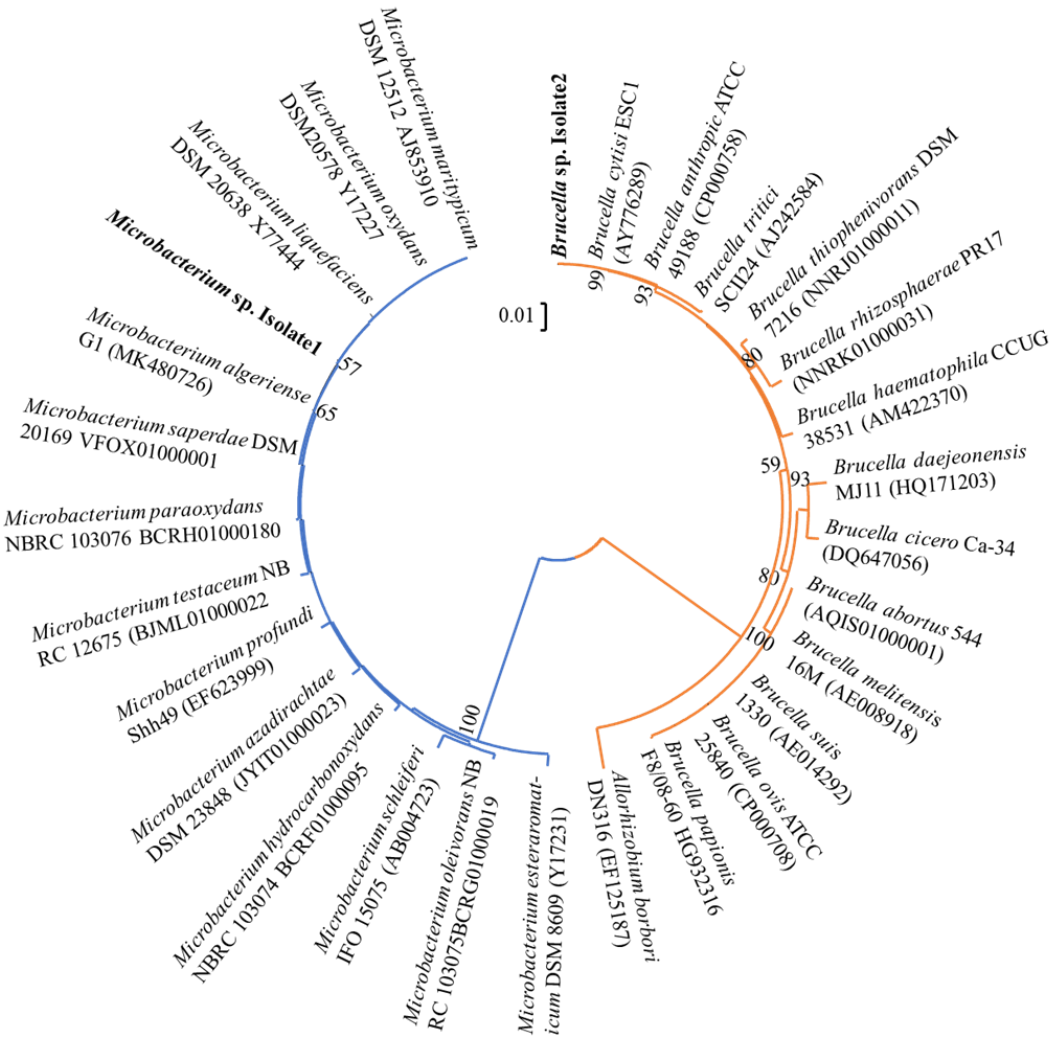

Figure S5. Phylogenetic tree of the isolated highly efficient degrading bacteria that do not actively participate in soil PHE degradation (Isolate1 and Isolate2). Neighbor-joining tree based on 16S ribosomal RNA (rRNA) gene sequences showing the phylogenetic positions of these strains and representatives of other related taxa. Bootstrap values (expressed as percentages of 1200 replications) > 50% are shown at the branch points. The bar indicates 0.01 substitutions per nucleotide position.

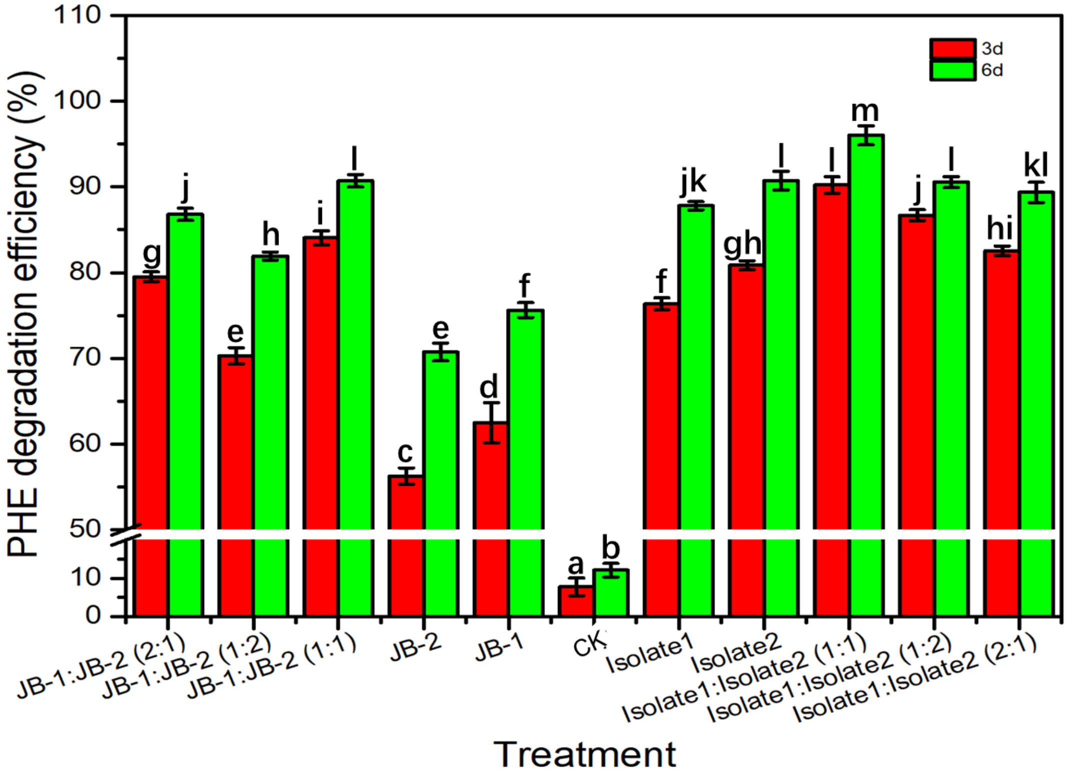

Figure S6. PHE degradation efficiency of different strains (including strains JB-1 and JB-2 selected by RACS, and highly efficient degrading bacteria that do not actively participate in PHE degradation (Isolate1 and Isolate2)) and their constructed functional microbial consortia in MM medium.

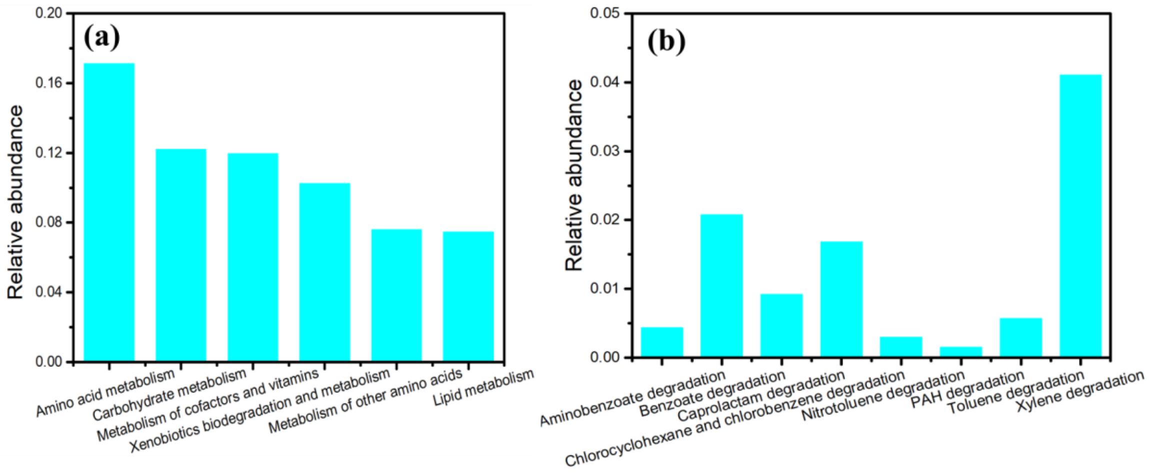

Figure S7. Functional analysis of genomic sequencing of sorted cells incorporating ^{13}C from the ^{13}C-PHE characterized by RACS based on KEGG pathway database. Relative abundance of 6 functional metabolism categories (a), and 8 functional categories related to the xenobiotics biodegradation and metabolism (b).

Table S1. Concentrations of petroleum content and soil characteristics. Data are means of three replicates.

**Soil characteristics**	**Content**
Clay	6.0%
Silt	29.3%
Sand	64.7%
pH	7.8
Total nitrogen	0.9 g/kg
Total phosphorus	0.5 g/kg
Total potassium	17.2 g/kg
Organic matter	29.2 g/kg
Soluble salts	2.8%
Petroleum	618.5 mg/Kg

Table S2. PHE degradation efficiency in soil after 3 and 6 days of incubation. Data are means of three replicates.

**Day**	**Sterile control**	**^{12}C-PHE treatment**
0	< 1.2%	< 1.0%
3	9.1-11.5%	72.7-76.1%
6	11.3-14.7%	> 98.6%

Table S3. Primers used for the PCR of 16S rRNA genes.

Target	Primer	Sequence (5’-3’)
16S rRNAs	515F	GTGCCAGCMGCCGCGGTAA
	806R	AACGCACGCTAGCCGGACTACVSGGGTATCTAAT
	27F	AGAGTTTGATCMTGGCTCAG
	1492R	TACGGYTACCTTGTTACGACTT

Table S4. PHE-degrading microorganisms isolated under indoor culture conditions and their PHE degradation efficiency in MM medium after 6 days of incubation.

**Strains**	**lineage**	**PHE degradation efficiency (%)**
Isolate1	*Microbacterium* sp.	87.8
Isolate2	*Brucella* sp.	90.7
Isolate3	*Gordonia* sp.	75.2
Isolate4	*Pseudoxanthomonas* sp.	78.5
Isolate5	*Sphingomonas* sp.	77.9
Isolate6	*Brucella* sp.	85.1
Isolate7	*Pseudoxanthomonas* sp.	76.8
Isolate8	*Rhodococcus* sp.	84.3
Isolate9	*Microbacterium* sp.	86.7
Isolate10	*Sphingomonas* sp.	80.4
Isolate11	*Sphingobacterium* sp.	82.6
Isolate12	*Ralstonia* sp.	84.2

Table S5. The information of the assembled bins from the RACS-sorted cells.

Bin	Completeness (%)	Contamination (%)	GC	lineage
Bin1	70.6	0	0.68	d__Bacteria;p__Proteobacteria;c__Gammaproteobacteria;o__Burkholderiales;f__Burkholderiaceae;g__Achromobacter
Bin3	76.1	2.54	0.68	d__Bacteria;p__Proteobacteria;c__Gammaproteobacteria;o__Pseudomonadales;f__Pseudomonadaceae;g__Pseudomonas

Table S6. Components of the modified minimal medium (a), vitamin stock solution (b) and mineral stock solution (c).

(a)

**Components**	**Concentration**
KH_2PO_4	2.5 g/L
NH_4 NO_3	3.0 g/L
$K_2HPO_4 \cdot 12H_2O$	2.0 g/L
$MgSO_4 \cdot 7H_2O$	0.2 g/L
$FeSO_4 \cdot 7H_2O$	0.1 g/L
Vitamin stock solution	10.0 ml/L
Mineral stock solution	10.0 ml/L
Streptomycin	1.0 mg/L
L-lactate	50.0 mM

(b)

**Components**	**Concentration**
Vitamin B1	5.0 mg/L
Vitamin B2	5.0 mg/L
Vitamin B6	5.0 mg/L
Vitamin B3	5.0 mg/L
Vitamin B7	5.0 mg/L
Vitamin B12	0.1 mg/L
Retinol	5.0 mg/L
Lipoic acid	5.0 mg/L

(c)

Components	Concentration
$CaCl_2$	5.0 mg/L
$CuSO_4$ $5H_2O$	0.4 mg/L
$CoCl_2$ $6H_2O$	2.0 mg/L
$NiCl_2$ $6H_2O$	2.0 mg/L
ZnSO4 ·7H2O	4.0 mg/L
NaCl	1.0 mg/L
Na2MoO4	2.0 mg/L
