# Supplementary material for: Microbial consortium assembly and functional analysis via isotope labelling and single-cell manipulation of polycyclic aromatic hydrocarbon degraders
Source: ISME J. 2024 Jun 24;18(1):wrae115. doi: 10.1093/ismejo/wrae115 (PMC11256997; doi:10.1093/ismejo/wrae115)
Supplement: non-data_SIP_wrae115 [file non-data_sip_wrae115.pdf]

Supplementary Materials for

Microbial consortium assembly and functional analysis via isotope labelling and single-cell manipulation of polycyclic aromatic hydrocarbon degraders

Jibing Li[a,b], Chunling Luo[a,b,*], Xixi Cai[c], Dayi Zhang[d,e], Guoqing Guan [a,b], Bei Li[f,g], Gan Zhang[a,b]

[a]State Key Laboratory of Organic Geochemistry and Guangdong-Hong Kong-Macao Joint Laboratory for Environmental Pollution and Control, Guangzhou Institute of Geochemistry, Chinese Academy of Sciences, Guangzhou 510640, China

[b]University of Chinese Academy of Sciences, Beijing 100039, China

[c]Guangdong Key Laboratory of Ornamental Plant Germplasm Innovation and Utilization, Environmental Horticulture Research Institute, Guangdong Academy of Agricultural Sciences, Guangzhou 510640, China

[d]Key Laboratory of Groundwater Resources and Environment, Ministry of Education, Jilin University, Changchun 130012, China

[e]College of New Energy and Environment, Jilin University, Changchun 130021, China

[f]State Key Lab of Applied Optics, Changchun Institute of Optics, Fine Mechanics and Physics, Chinese Academy of Sciences, 130033, Changchun, China

[g]HOOKE Instruments Ltd., 130033, Changchun, China

*Corresponding author: Dr. Chunling Luo

E-mail: clluo@gig.ac.cn Tel.: +86-20-85290290; Fax: +86-20-85290706

Soil characteristics

Soil texture was measured using the hydrometer method[1]. Soil total N (TN) and organic matter were measured with a Virio TOC analyser (Elementar, Germany).

DNA ultracentrifugation and real-time quantitative PCR

DNA extraction from each sample was performed using the PowerSoil DNA Isolation Kit (MO BIO, Carlsbad, CA) according to the manufacturer's instructions. Subsequently, the DNA obtained from the ^{12}C_PHE and ^{13}C_PHE microcosms was employed for CsCl gradient ultracentrifugation, following established protocols.[2, 3] Briefly, approximately 5 μg of DNA was mixed with a tris EDTA/CsCl solution at a buoyancy density (BD) of approximately 1.77 g/ml and loaded into Quick-Seal polyallomer tubes (13×51 mm, 5.1 mL, Beckman Coulter, Pasadena, CA, USA). Ultracentrifugation was carried out in a Beckman Coulter L-100XP ultracentrifuge at 47,500 rpm for 48 hours at 20 ℃. Subsequently, 14 fractions were separated from each tube using a fraction recovery system (Beckman Coulter). The BD values of the separated DNA samples were determined using an AR200 digital refractometer, and DNA purification was achieved through glycogen-assisted ethanol precipitation.[1, 4]

For amplification of bacterial 16S rRNA genes in all DNA fractions from the ^{12}C_PHE and ^{13}C_PHE microcosms, a specific primer set of 515F/806R was employed. The qPCR reaction system was set up as described previously.[3, 5] After gel purification, PCR products were cloned into the pGEM-T plasmid vector and subjected to sequencing. To generate a standard curve, 10-fold serial dilutions of recombinant sequences extracted from *E. coli* were utilized. The qPCR program consisted of an initial denaturation at 94° C for 3 minutes, followed by 40 cycles of 94℃ for 15 seconds, 55℃ for 30 seconds, and 72℃ for 30 seconds. Each DNA sample was amplified in triplicate. Based on the relationship between BD values and 16S rRNA gene abundance in each fraction from SIP microcosms (Figure 1), the DNA fraction with a buoyant density of 1.7415 g/m was designated as the "heavy" DNA (^{13}C-DNA) from the ^{13}C_PHE microcosms.

For amplicon sequencing, the hypervariable V4 region was amplified from DNA extracted from

all treatments, as well as from the separated DNA of the ^{12}C_PHE and ^{13}C_PHE treatments, using the 515F and 806R primer set (Table S3). PCR was conducted following our previous study.[6, 7] After purification and quantification, PCR products were sequenced on an Illumina MiSeq sequencer using 2 × 250 bp PE technology. Paired-end 16S rDNA reads were merged using FLASH v1.2.11. Low-quality reads (length < 200 bp, >2 'N' bases, average quality < 30) were filtered using a custom Python script. Subsequently, sequences were processed and analyzed using Quantitative Insights into Microbial Ecology (QIIME2, pipeline v2019.10.0)[8]. DADA2 was employed to obtain amplified sequence variants (ASVs) [9]. Taxonomic assignments of the 16S rRNA gene were performed utilizing the SILVA (release_138) database [10]. Human contamination (Blastn E-value threshold ≤ 10^{-5}, bitscore ≥ 50, percent identity ≥ 75%) was further excluded by aligning reads to the human reference genome (build 37) using bowtie2 (version 2.1.0).[11]

Identification and isolation of ^{13}C cells by RACS

To identify and sort the active degrading bacterial cells, samples from the ^{13}C_PHE microcosms were utilized for RACS, while those from the ^{12}C_PHE microcosms served as control samples. The bacterial cells present in the initial samples at t = 0 days were employed as a reference point for a RACS benchmarking experiment. Initially, 3 mL of the sample was transferred into a centrifuge tube and placed in an ice block, followed by sonication at 300 mW for 1 minute, with a 1-second pause for every second of sonication. To eliminate any impurities that could potentially interfere with the acquisition of Raman spectra, the samples were centrifuged at 2000 g for 5 minutes. The supernatants were collected to isolate and clean the bacterial cells, which were then centrifuged at 5000 g for 5 minutes and subjected to four washes with deionized water. The resulting microbial cell pellet was subsequently resuspended in deionized water, and 2 μL of the cell suspension was deposited onto a sorting chip (metal-coated ejection chip, HOOKE Instruments Ltd., Changchun, China), air-dried at room temperature before Raman spectral acquisition. Spectroscopy via stimulated Raman scattering (SCRS) was conducted using a 532-nm

neodymium-doped yttrium aluminum garnet laser (Laser Quantum, Bedford, MA, USA) with a 300-grooves/mm diffraction grating. Raman spectra were captured in the range of 400–2500 cm^{-1}, employing a laser power of 5 mW and an acquisition time of 5 seconds. The acquired spectral data were subjected to preprocessing using LabSpec 6 software (Horiba) to perform baseline correction and vector normalization.

In cases where bacterial cells utilize isotopically labeled substrates and synthesize macromolecular markers, such as phenylalanine, heavier isotopic atom replace lighter atom, leading to changes in its bond vibration and a shift in its wavenumber to lower position.[12] According to prior studies, the Raman band position for unlabeled bacterial cells is approximately 1001–1003 cm^{-1} for phenylalanine.[4, 12] In the case of functional bacterial cells incorporating ^{13}C, this Raman band exhibit significant redshift of approximately -37 cm^{-1}. Consequently, the positions of the Raman band detected via SCRS in functional bacterial cells that have assimilated ^{13}C-PHE were analysed to establish a relationship between the redshift and ^{13}C assimilation in the ^{13}C_PHE microcosms.

Isolation of highly efficient degrading bacteria that do not actively participate in PHE degradation and the construction of a functional microbial consortium.

To compare the bioremediation efficacy against the *in-situ* FMC mentioned above, we employed conventional isolation techniques to procure two proficient PHE-degrading strains, designated as Isolate1 and Isolate2. It is noteworthy that although we identified over ten proficient PHE-degrading microorganisms, including Isolate1 and Isolate2, they do not actively contribute to PHE degradation in oil-contaminated soil (Table S4). These strains were cultivated and isolated from oil-contaminated soil employing MM medium supplemented with PHE (50 mg L−1) as the sole carbon source, using the spread plate method on MM agar medium.[13] Briefly, to enrich and isolate the highly efficient PHE-degrading bacteria, a 5.0 g sample of contaminated soil was introduced into 100 mL of MM Medium containing 50 mg/L of PHE.[14] This mixture was subjected to incubation in the dark at 28 ℃ while agitating at 180 rpm for duration of one week. Subsequently, 5 mL of the enriched solution was aseptically transferred to fresh enriched culture medium, and this process was repeated thrice. Following this enrichment phase, the culture

medium was subjected to serial dilution and plating onto MM plates with 50 mg/L of PHE. Plates were then incubated at 28 ℃, and individual colonies were meticulously selected and transferred to fresh plates for further purification and identification.

For their identification, we employed universal primers 27F and 1492R for PCR amplification. The PCR reaction mixture consisted of 25 μL, including 12.5 μL of rTaq premixed buffer (TaKaRa), 0.5 μL of forward primer (10 mmol/L), 0.5 μL of reverse primer (10 mmol/L), and 1 μL of template DNA. Deionized water was added to adjust the final volume to 10.5 μL. The PCR amplification was conducted with the following conditions: an initial denaturation at 96 ℃ for 6 minutes, followed by denaturation at 95 ℃ for 45 seconds, annealing at 56 ℃ for 45 seconds, and extension at 72 ℃ for 90 seconds (30 cycles). The reaction concluded with a final extension at 72 ℃ for 10 minutes. PCR products from bacterial samples were confirmed through agarose gel electrophoresis to ensure the appropriate size of the amplification fragments. Subsequently, the PCR products underwent sequencing by BGI. Based on the BLAST results, we successfully identified two highly efficient PHE-degrading bacterial strains, namely Isolate1 and Isolate2.

The degradation experiments for these bacterial strains and the constructed bacterial consortia were conducted under the identical conditions as those employed for RACS-sorted microbes. In summary, Isolate1 and Isolate2 were cultured in darkness at 30 ℃ in MM medium with 50 mg/L PHE for 4 days under continuous agitation at 180 rpm. Enriched cultures were transferred to sterile centrifuge tubes, centrifuged at 4000 × g for 10 minutes to obtain cell pellets, and subsequently resuspended in phosphate-buffered saline (PBS). These suspensions, containing either Isolate1 or Isolate2, or a combination of both, were introduced into 150 mL brown glass bottles with 50 mg/L PHE. Inoculations were configured as monocultures or co-cultures (ratios: CFU 1:1, 1:2, or 2:1), with non-inoculated treatments as the blank control. Experiments were conducted in triplicate, and samples were collected at days 0, 3, and 6 for destructive sampling to extract PHE.

References

1. Bao, J. et al. New insight into the mechanism underlying the effect of biochar on phenanthrene degradation in contaminated soil revealed through DNA-SIP. *J. Hazard. Mater.* **438**, 129466 (2022).
2. Li, J. et al. Novel bacteria capable of degrading phenanthrene in activated sludge revealed by stable-isotope probing coupled with high-throughput sequencing. *Biodegradation* **28**, 423-436 (2017).
3. Li, J. et al. The catabolic pathways of in situ rhizosphere PAH degraders and the main factors driving PAH rhizoremediation in oil-contaminated soil. *Environ. Microbiol.* **23**, 7042-7055 (2021).
4. Li, J., Zhang, D., Li, B., Luo, C. & Zhang, G. Identifying the Active Phenanthrene Degraders and Characterizing Their Metabolic Activities at the Single-Cell Level by the Combination of Magnetic-Nanoparticle-Mediated Isolation, Stable-Isotope Probing, and Raman-Activated Cell Sorting (MMI–SIP–RACS). *Environ. Sci. Technol.* **56**, 2289-2299 (2022).
5. Cai, X.X., Li, J.B., Guan, F.Y., Luo, X.S. & Yuan, Y. Unveiling metabolic characteristics of an uncultured Gammaproteobacterium responsible for in situ PAH biodegradation in petroleum polluted soil. *Environ. Microbiol.* **23**, 7093-7104 (2021).
6. Luo, C. et al. Toward a More Comprehensive Understanding of Autochthonous Bioaugmentation (ABA): Cases of ABA for Phenanthrene and Biphenyl by Ralstonia sp. M1 in Industrial Wastewater. *ACS ES&T Water* **1**, 1390-1400 (2021).
7. Li, J. et al. Stable-isotope probing enabled cultivation of the indigenous strain Ralstonia sp. M1 capable of degrading phenanthrene and biphenyl in industrial wastewater. *Appl. Environ. Microbiol.* **85**, AEM.00511-00519 (2019).
8. Bolyen, E. et al. Reproducible, interactive, scalable and extensible microbiome data science using QIIME 2. *Nat. Biotechnol.* **37**, 852-857 (2019).
9. Callahan, B.J. et al. DADA2: High-resolution sample inference from Illumina amplicon data. *Nat. Methods* **13**, 581-583 (2016).
10. Quast, C. et al. The SILVA ribosomal RNA gene database project: improved data processing and web-based tools. *Nucleic Acids Res.* **41**, D590-596 (2013).
11. Langmead, B., Trapnell, C., Pop, M. & Salzberg, S.L. Ultrafast and memory-efficient alignment of short DNA sequences to the human genome. *Genome Biol.* **10**, R25 (2009).
12. Wang, Y., Huang, W.E., Cui, L. & Wagner, M. Single cell stable isotope probing in microbiology using Raman microspectroscopy. *Curr. Opin. Biotechnol.* **41**, 34-42 (2016).
13. Li, J. et al. Paenibacillus guangzhouensis sp. nov., an Fe (III)-and humus-reducing bacterium from a forest soil. *Int. J. Syst. Evol. Microbiol.* **64**, 3891-3896 (2014).
14. Li, J. et al. Biodegradation of Phenanthrene in Polycyclic Aromatic Hydrocarbon-Contaminated Wastewater Revealed by Coupling Cultivation-Dependent and -Independent Approaches. *Environ. Sci. Technol.* **51**, 3391-3401 (2017).
